# Pharmacological Targeting of CXCR4 Attenuates Sepsis-Induced Intestinal Injury by Suppressing NLRP3/GSDMD-Mediated Pyroptosis

**DOI:** 10.1007/s10753-026-02494-7

**Published:** 2026-04-02

**Authors:** Hua Xu, Shuying Yang, Hongjie Li, Dingbin Liu, Hongmei Gao

**Affiliations:** 1 Department of Intensive Care Unit, Key Laboratory for Critical Care Medicine of the Ministry of Health, Emergency Medicine Research Institute, Tianjin First Central Hospital, Nankai University, Tianjin, 300192 China; 2 State Key Laboratory of Medicinal Chemical Biology, Research Center for Analytical Sciences, Tianjin Key Laboratory of Biosensing and Molecular Recognition, College of Chemistry, Nankai University, Tianjin, 300071 China

**Keywords:** Sepsis, Intestinal barrier, Pyroptosis, CXCR4, AMD3100

## Abstract

Sepsis-induced intestinal barrier dysfunction is a critical driver of multiple organ failure and associated mortality. Although pyroptosis contributes to sepsis pathogenesis, the upstream receptors that trigger this process and constitute viable therapeutic targets remain elusive. Here, we identify C-X-C chemokine receptor type 4 (CXCR4) as a key upstream regulator of intestinal epithelial pyroptosis during septic injury. In a murine cecal ligation and puncture (CLP) model, sepsis robustly upregulated CXCR4 expression, which subsequently induced NLRP3 upregulation, caspase-1 activation, and generation of the gasdermin D N-terminal fragment (GSDMD-NT). Pharmacological inhibition of CXCR4 with the clinically approved antagonist AMD3100 (Plerixafor) significantly attenuated intestinal injury, restored the expression of tight junction proteins (ZO-1 and occludin), and suppressed pyroptotic markers. Conversely, CXCR4 agonism with NUCC-390 exacerbated intestinal damage. Mechanistically, CXCR4 activation enhanced NF-κB p65 phosphorylation, thereby propelling NLRP3-dependent GSDMD cleavage. Genetic knockdown of GSDMD abolished NUCC-390-induced pyroptosis and barrier impairment without affecting NF-κB activation, confirming GSDMD as the essential downstream executor. Furthermore, pharmacological inhibition of NF-κB abrogated the exacerbation of intestinal injury, barrier dysfunction, and pyroptosis induced by CXCR4 activation, definitively establishing NF-κB as a necessary signaling mediator within this CXCR4-driven pathway. These findings establish the CXCR4/NF-κB/NLRP3/GSDMD axis as a novel and therapeutically targetable pathway in septic intestinal injury. Importantly, we demonstrate that the FDA-approved CXCR4 antagonist AMD3100 represents a promising preclinical therapeutic candidate, exhibiting dual anti-inflammatory and anti-pyroptotic efficacy. Our work thus provides a mechanistically grounded rationale for further investigation of its repurposing potential in sepsis.

**Supplementary Information:**

The online version contains supplementary material available at 10.1007/s10753-026-02494-7.

## Introduction

Sepsis, defined as life-threatening organ dysfunction resulting from a dysregulated host response to infection, remains a leading cause of mortality in intensive care units worldwide [1, 2]. Despite significant advances in antimicrobial therapy and supportive care, mortality rates—particularly among patients who progress to multiple organ dysfunction syndrome (MODS)—remain unacceptably high [1, 3]. Importantly, the gastrointestinal tract is not merely a passive target in sepsis; rather, it functions as a pathogenic “engine” whose barrier failure drives systemic inflammation, bacterial translocation, and subsequent secondary organ injury [4, 5]. Therefore, preserving intestinal barrier integrity represents a pivotal therapeutic objective for improving survival in sepsis.

The intestinal epithelium constitutes a dynamic barrier that selectively restricts luminal pathogens, toxins, and antigens from entering the systemic circulation [6]. Sepsis-induced compromise of epithelial integrity—primarily through excessive cell death, junctional disruption, or epithelial detachment—results in pathological hyperpermeability. This facilitates bacterial and toxin translocation, thereby fueling a vicious cycle of inflammation and multi-organ failure [6, 7]. Consequently, elucidating the mechanisms underlying septic barrier dysfunction and developing targeted barrier-protective agents represent urgent and unmet clinical needs.

Pyroptosis is an inflammatory, caspase-dependent form of programmed cell death that releases potent cytokines such as IL-1β and IL-18, thereby amplifying inflammation [8, 9]. Although GSDMD-mediated pyroptosis exerts antimicrobial effects [10–12], its uncontrolled activation in the intestinal epithelium represents a double-edged sword: it disrupts barrier function while simultaneously exacerbating systemic inflammation [13, 14]. Consistent with emerging evidence [15–17], our CLP model confirms that septic intestinal injury coincides with epithelial pyroptosis. Nevertheless, the key upstream receptors that initiate pathological NLRP3/GSDMD hyperactivation in the septic gut remain elusive.

Transcriptomic profiling of CLP-injured intestines revealed a striking upregulation of C-X-C chemokine receptor type 4 (CXCR4) (log_2_FC = 3.25, Q = 0.0101). CXCR4, a ubiquitously expressed G-protein-coupled receptor (GPCR), modulates inflammatory responses via NF-κB in diverse pathological contexts [18–20]. However, its role in regulating pyroptosis during sepsis-induced intestinal barrier failure remains unknown, and its potential as a therapeutic target for epithelial protection has yet to be explored.

Here, we hypothesize that CXCR4 acts as a master upstream regulator driving NLRP3/GSDMD-mediated pyroptosis in the septic intestinal epithelium. Using integrated genetic (GSDMD knockdown) and pharmacological (CXCR4 antagonist AMD3100 / agonist NUCC-390) approaches, we demonstrate for the first time that: (1) CXCR4 activation is both necessary and sufficient to trigger epithelial pyroptosis and barrier disruption via the NF-κB/NLRP3/GSDMD axis; (2) GSDMD serves as the non-redundant terminal effector of CXCR4-mediated damage; (3) critically, the FDA-approved CXCR4 antagonist AMD3100 (Plerixafor) concurrently suppresses inflammation and pyroptosis, rescues barrier integrity, and thereby reveals its therapeutic potential while providing a clear mechanistic rationale for further investigation.

These findings collectively delineate the CXCR4/NF-κB/NLRP3/GSDMD axis as a central and druggable signaling pathway in sepsis-induced intestinal injury and highlight the repurposing potential of the FDA-approved CXCR4 antagonist AMD3100—offering a mechanistically grounded and promising strategy to improve outcomes in sepsis.

## Materials and Methods

### Experimental Animals

Male C57BL/6 mice (RRID: MGI: 2159769) were purchased from HuaFuKang Biotechnology Co., Ltd. [laboratory animal license: SCXK (Beijing) 2019-0008; Beijing, China]. Mice aged 6–8 weeks were housed in standard polypropylene cages with ad libitum access to autoclaved rodent chow and filtered water. All animals were acclimatized for at least 7 days prior to experiments under controlled environmental conditions: 12-hour light/dark cycle, ambient temperature of 22 ± 2 °C, and relative humidity at 55 ± 10%. Bedding was changed twice weekly to maintain hygiene.

### Ethics Statement

All animal procedures were performed in accordance with the ARRIVE guidelines (Animal Research: Reporting of In Vivo Experiments). The protocol was approved by the Animal Ethics Committee of Nankai University (Approval No. 2023-SYDWLL-000386).

### Cecal Ligation Perforation (CLP) Model

Mice were fasted for 12 h with free access to water prior to surgery. Under aseptic conditions, animals were anesthetized by intraperitoneal injection of pentobarbital sodium (50 mg/kg, Sigma-Aldrich P3761). A 1-cm midline laparotomy was performed to expose the cecum. Approximately the distal one-third of the cecum was ligated with 4 − 0 silk suture without obstructing intestinal continuity. Two through-and-through punctures were created using a 21-gauge needle, followed by gentle extrusion of fecal content. The abdomen was then closed in layers. Sham-operated control mice underwent an identical surgical procedure except for the ligation and puncture steps. Postoperatively, all mice received 1 mL of sterile saline subcutaneously (s.c.) for fluid resuscitation and sustained-release buprenorphine (1.0 mg/kg, s.c.) every 12 h for analgesia. For the initial experiment validating sepsis-induced intestinal dysfunction and pyroptosis, mice were randomly divided into CLP group (*n* = 6) and Sham group (*n* = 6).

### GSDMD-Knockdown Model

The GSDMD knockdown mouse model was generated via tail vein injection of recombinant adeno-associated virus (AAV) (1 × 10¹¹ vg/mouse in 100 µL PBS). The virus carried three tandem shRNA sequences targeting murine Gsdmd (shRNA-1: 5’-GCCATCGGCCTTTGAGAAAGT-3’; shRNA-2: 5’-GCTGCAGACAAAGGAGGAAGT-3’; shRNA-3: 5’-GGGATTGATGAGGAGGAATTA-3’) packaged in GV248 U6-MCS-CAG-EGFP vector. A control virus expressing a scramble shRNA sequence (5′-CGCTGAGTACTTCGAAATGTC-3′) was injected into control mice. All AAV preparations were produced by Shanghai Genechem Biotechnologies Co., Ltd (Shanghai, China). Four weeks post-injection, successful knockdown was validated by RT-qPCR (primers: F 5’-GCTCAGTCTCCTGTCAGATGG-3’, R 5’-CAAGCCTTCACCTCAGCATACA-3’; β-actin as reference) and western blotting. To evaluate the effect of GSDMD knockdown on CLP-induced intestinal dysfunction and pyroptosis, mice were randomly allocated into four groups (*n* = 6 per group): Sham, Sham+GSDMD-KD, CLP and CLP+GSDMD-KD. Among them, the Sham and CLP group mice were injected with the scramble-shRNA control virus, the Sham+GSDMD-KD and CLP+GSDMD-KD group mice were injected with the *Gsdmd*-targeting shRNA virus. All groups then underwent the CLP or sham procedure as described.

### CXCR4 Pharmacological Intervention Models

For CXCR4 inhibition, AMD3100 (Sigma-Aldrich, Cat# A5602) was dissolved in sterile 1× PBS (pH 7.4) and administered via subcutaneous injection (s.c.) at 5 mg/kg (in 100 µL) 1 h post-CLP [21]. For CXCR4 activation, NUCC-390 (GLPBio, Cat# GC39297) was prepared in PBS (26 mg/kg) and injected intraperitoneally (i.p.) at the same time point [22]. All injections were performed under brief isoflurane anesthesia, with control mice receiving PBS alone. Mice were randomly allocated to six groups (*n* = 6/group): Sham, Sham+AMD3100, Sham+NUCC-390, CLP, CLP+AMD3100, and CLP+NUCC-390. In rescue experiments, five additional groups (*n* = 6/group) were established: Sham, Sham+NUCC-390, CLP, CLP+NUCC-390, and CLP+NUCC-390 + GSDMD-KD (Gsdmd-knockdown C57BL/6 mice). All pharmacological interventions were performed following CLP procedures as described.

### NF-κB Inhibition Model

To mechanistically interrogate the role of NF-κB signaling downstream of CXCR4, we employed a pharmacological inhibition strategy using BAY11-7082 (Sigma-Aidrich, Cat# B5556-10MG), a specific and well-characterized inhibitor of NF-κB activation. For in vivo administration, BAY11-7082 was freshly dissolved in sterile, pyrogen-free 0.9% normal saline immediately before use. The solution was administered via intraperitoneal injection at a dose of 5 mg per kg body weight, with an injection volume of 100 µL per mouse, immediately upon completion of the CLP or sham surgical procedure. For this specific series of experiments, a separate cohort of age- and weight-matched mice was randomly allocated into the following five groups (*n* = 6/group): Sham, CLP, CLP+NUCC-390, CLP+ BAY11-7082, CLP+NUCC-390 + BAY11-7082. The CXCR4 agonist NUCC-390 was prepared and administered as described in Sect. 2.5. All pharmacological interventions were performed following CLP procedures as described.

### Functional Intestinal Permeability Assay

Gut barrier function was assessed in vivo using the FITC-dextran permeability assay. Mice were orally gavaged with 500 µL 22 mg/mL 4 kDa FITC-dextran (Yuanye Bio-Technology, Cat# S25807). Serum was separated by centrifugation. Fluorescence intensity was determined with excitation/emission wavelengths of 485/535 nm. A standard curve of FITC-dextran was prepared for each assay to calculate serum concentrations.

### Histopathological Analysis

Intestinal tissues were harvested 12 h post-CLP, fixed in 4% paraformaldehyde, and embedded in paraffin. Hematoxylin and eosin (H&E) staining was performed using standard protocols on 5-µm sections, and pathological scoring was conducted by two blinded investigators using the Chiu scoring system, as previously described.

### Enzyme-Linked Immunosorbent Assay (ELISA)

Intestinal tissue homogenates were centrifuged at 3000 rpm for 10 min at 4℃. Supernatants were analyzed for intestinal fatty acid binding protein (I-FABP) (FANKEW, Cat#F2044-B), TNF-α (FANKEW, Cat#F2132-A), IL-1β (FANKEW, Cat#F2040-A), IL-6 (FANKEW, Cat#F2163-A), and IL-18 (FANKEW, Cat#F2169-A) using commercial ELISA kits from Shanghai Kexing Trading Co., Ltd (FANKEW, Shanghai, China), according to the manufacturers’ protocols. Absorbance was measured at 450 nm with a microplate reader (PERLONG, Beijing, China). Standard curves were generated for each assay.

### Immunofluorescence Staining

Intestinal barrier integrity was assessed by immunofluorescence staining for tight junction proteins (ZO-1, occludin). Deparaffinized sections were stained with anti-ZO-1 (1:200, Proteintech, Cat#21773-1-AP) and anti-occludin (1:200, Abcam #ab216327) antibodies. Nuclei were counterstained with DAPI (Sigma-Aldrich, Cat#D9542). Stained slides were scanned using a PANNORAMIC SCAN II whole-slide imaging system (3DHISTECH, Hungary). Fluorescence signals were captured under standardized exposure settings for all samples. Image analysis was performed using SlideViewer 2.5 (3DHISTECH).

### Western Blotting

Tissues were lysed in RIPA buffer containing protease/phosphatase inhibitors. Protein concentrations were determined by BCA assay. Equal amounts were separated by SDS-PAGE and transferred to PVDF membranes. The membranes were probed overnight at 4 °C with primary antibodies against: NLRP3 (1:1000, BOSTER, Cat# BA3677), GSDMD (1:1000, CST, Cat# 39754 S), Caspase-1 (1:1000, CST, Cat# 83383), ZO-1 (1:25000, Proteintech, Cat#21773-1-AP), occludin (1:25000, Proteintech, Cat#66378-1-IG), Phospho-NF-κB p65 (1:1000, CST, Cat# 3033), and GAPDH (1:3000, ZSGB-BIO, Cat# TA-08). Goat anti-Rabbit IgG (1:5000, ZSGB-BIO, Cat# ZB-2301) and Goat anti-Mouse IgG (1:5000, ZSGB-BIO, Cat# ZB-2305) were used for detection with ECL substrate. Band intensities were quantified using ImageJ.

### Co-localization Immunofluorescence

Paraffin-embedded intestinal Sect. (5 μm) were deparaffinized, rehydrated, and subjected to antigen retrieval in EDTA buffer (pH 9.0) using microwave heating. After blocking with 10% goat serum, sections were incubated overnight at 4 °C with primary antibodies against: Anti-GSDMD (1:200, Proteintech, #20770-1-AP) and Anti-Cytokeratin19 (1:200, Proteintech, #10712-1-AP). A species-matched secondary antibody Goat anti-rabbit IgG-Cy3 (1:50, ZSGB-BIO, #ZF-0316) was applied. Nuclei were counterstained with DAPI (Sigma-Aldrich, Cat#D9542). Stained slides were scanned using a PANNORAMIC SCAN II whole-slide imaging system (3DHISTECH, Hungary). Fluorescence signals were captured under standardized exposure settings for all samples. Co-localization analysis was performed using SlideViewer 2.5 (3DHISTECH).

### RNA Sequencing and Bioinformatics

Total RNA was extracted using TRIzol (Invitrogen, Life Technologies, United States) according to the manufacturer’s protocol. The concentration, purity, and integrity of the RNA samples were assessed using a Nanodrop 2000 spectrophotometer and the RNA 600 Nano Assay Kit for the 2100 Bioanalyzer system (Agilent Technologies, CA, USA). Libraries were constructed and sequenced using the DNBSEQ platform by BGI technologies (BGI-Shenzhen, China). The seqFITC dextranuence data were filtered with SOAPnuke and the cleaned data were stored in a FASTQ format and subsequently imported into the Dr. Tom bioinformatics analysis system. Differential expression analysis was performed with DESeq2 (|log_2_FC| ≥ 1, Q value < 0.05). Gene ontology (GO) and KEGG pathway analyses were conducted using clusterProfiler. The level of significance of the terms and pathways was corrected using p values with a rigorous threshold (*P* value ≤ 0.05).

### Immunohistochemistry (IHC)

After antigen retrieval and endogenous peroxidase blocking, sections were incubated with anti-CXCR4 (1:200, BOSTER, Cat# PA1237) overnight at 4 °C. Signal amplification was performed using an enhancement system (37 °C, 20 min each for reaction enhancer and polymer), followed by DAB development with microscopic monitoring. Sections were counterstained with hematoxylin, dehydrated, and mounted. Stained slides were scanned using a PANNORAMIC SCAN II whole-slide imaging system (3DHISTECH, Hungary). Image analysis was performed using SlideViewer 2.5 (3DHISTECH).

### Statistical Analysis

Continuous data are presented as mean ± standard deviation (SD). Between-group comparisons were performed using unpaired two-tailed Student’s t-tests for comparisons between two groups only, one-way ANOVA with Tukey’s post-hoc test for comparisons among three or more groups. Statistical significance was set at *P* < 0.05. All statistical analyses were performed using R (version 4.5.0, R Foundation for Statistical Computing) and GraphPad Prism (version 8.0, GraphPad Software). Data are presented throughout the manuscript as dot-plotted bar graphs to show individual data points and illustrate distribution. Final figures were prepared using GraphPad Prism, Adobe Photoshop, and Adobe Illustrator 2024.

## Results

### CLP-Induced Intestinal Barrier Dysfunction and Pyroptosis in Septic Mice

**Fig. 1:**
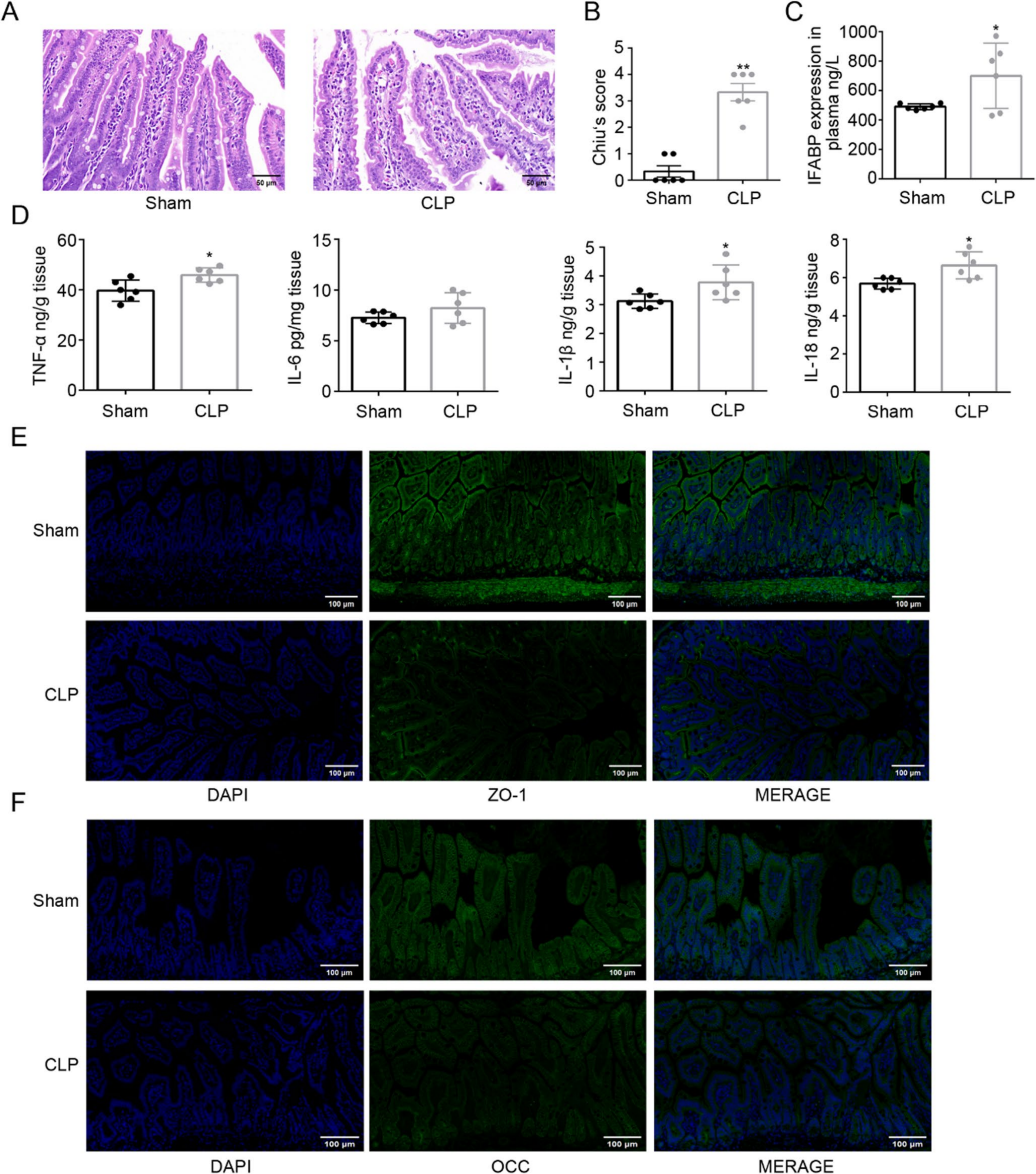
CLP-induced intestinal barrier dysfunction and pyroptosis. **A** H&E staining of intestinal sections (scale bar: 50 μm) showing mucosal erosion and neutrophil infiltration in CLP mice compared to Sham controls. **B** Chiu's pathological scores (**P < 0.01 vs. sham group). **C** Elevated I-FABP in CLP intestinal homogenates (*P < 0.05 vs. sham group). **D** Pro-inflammatory cytokine (TNF-α, IL-1β, IL-18: *P < 0.05 vs. sham group; IL-6: *P* >0.05 vs. sham group) by ELISA. **E**, **F** Disrupted ZO-1/occludin continuity (green) by immunofluorescence (scale bar: 100 μm). Nuclei are counterstained with DAPI (blue). Data are presented as mean ± SD, *n* = 6 mice per group. Statistical significance was determined by unpaired two tailed Student’s t test

Histopathological examination of intestinal tissues at 12 h post-CLP revealed severe architectural disruption, characterized by goblet cell depletion, mucosal erosion, and extensive inflammatory cell infiltration such as lymphocytes and neutrophils, contrasting with the intact morphology observed in Sham group (Fig. 1A). Quantitative assessment using Chiu’s score confirmed significantly greater intestinal injury in CLP mice compared to Sham group (*P* < 0.01; Fig. 1B).

Intestinal barrier dysfunction was further evidenced by elevated levels of intestinal fatty acid-binding protein (I-FABP) in CLP mice (*P* < 0.05; Fig. 1C), indicative of enterocyte damage. Pro-inflammatory cytokine analysis revealed significant upregulation of TNF-α, IL-1β, and IL-18 in CLP mice compared to Sham group (*P* < 0.05 for all), whereas IL-6 levels were unaltered (*P* > 0.05; Fig. 1D), underscoring robust intestinal inflammation.

Immunofluorescence staining revealed a marked reduction in the expression and discontinuous distribution of tight junction proteins ZO-1 and occludin in CLP mice, in contrast to the continuous linear pattern observed in Sham group (Fig. 1E, F). These findings collectively highlight the compromised tight junction integrity following CLP.

### GSDMD-Mediated Pyroptosis Contributes to Septic Intestinal Injury

**Fig. 2:**
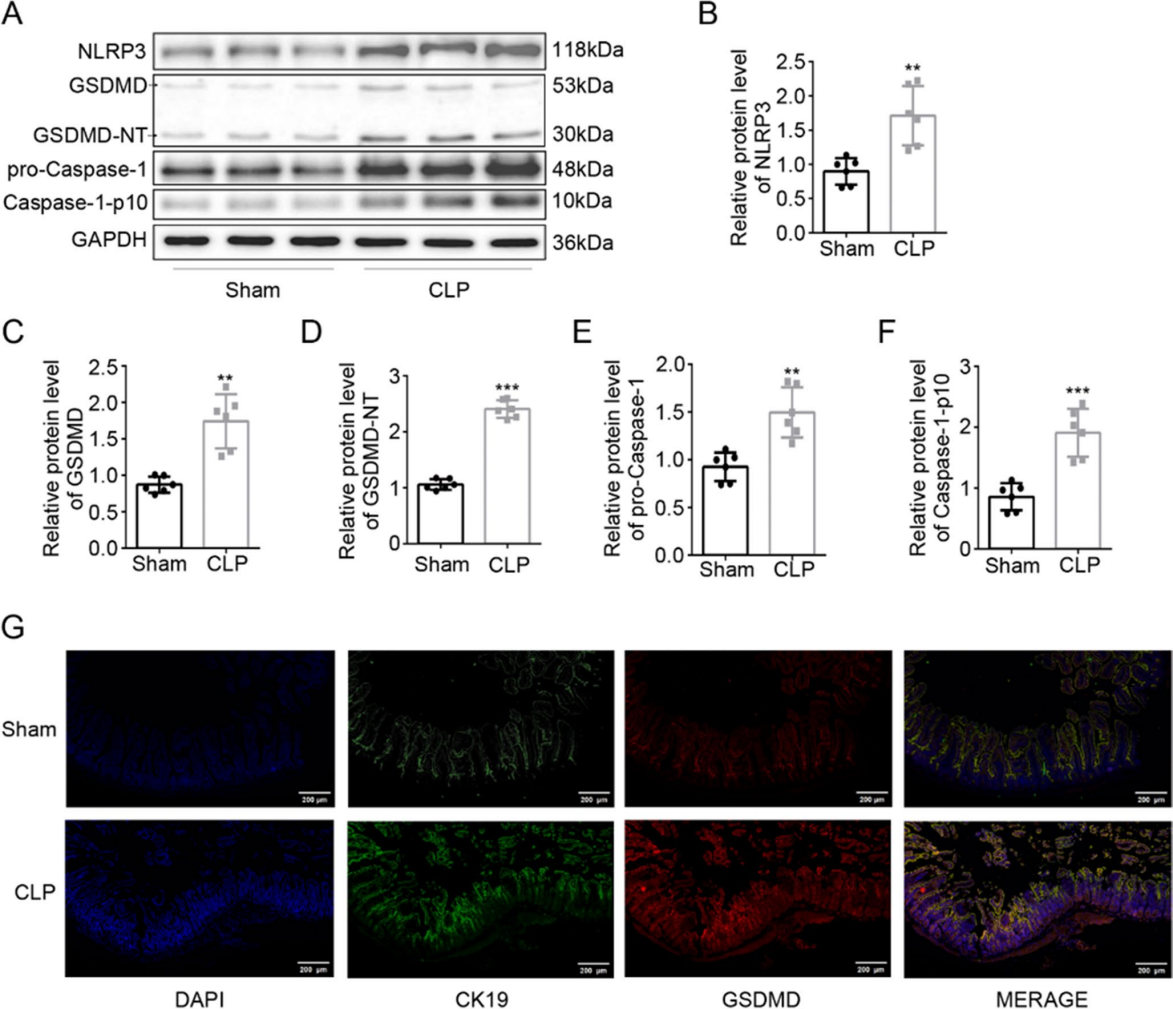
GSDMD-dependent pyroptosis exacerbates intestinal injury. **A**, **B** Western blot and quantification of pyroptosis markers in CLP mice: NLRP3 (**P < 0.01 vs. sham group), caspase-1-p10 (***P < 0.001 vs. sham group), GSDMD-NT (***P < 0.001 vs. sham group). **C** GSDMD (red) co-localization with epithelial CK19 (green) by immunofluorescence (scale bar: 200 μm; DAPI: blue). Data are expressed as the mean ± SD; *n* = 6 mice per group. Statistical significance was determined by unpaired two tailed Student’s t test

To elucidate the role of pyroptosis in sepsis-induced intestinal injury, we systematically analyzed the activation of pyroptotic signaling pathways. Western blot analysis revealed significant upregulation of key pyroptotic components in CLP mice compared to Sham mice, including NLRP3 (*P* < 0.01), the active caspase-1 fragment (caspase-1-p10, *P* < 0.001), and GSDMD-N-terminal fragments (GSDMD-NT, *P* < 0.001) (Fig. 2A-F). These molecular alterations confirmed the activation of canonical pyroptotic pathways in septic intestinal tissues.

Immunofluorescence co-localization studies demonstrated significant accumulation of GSDMD with the enterocyte marker CK19 in CLP mice, with prominent GSDMD specifically localized to intestinal epithelial cells (Fig. 2G). The spatial association of GSDMD activation with epithelial cells, combined with biochemical evidence of inflammasome assembly (NLRP3) and effector cleavage (caspase-1-p10 and GSDMD-NT), establishes pyroptosis as a key mechanism of epithelial cell death during sepsis.

### GSDMD Knockdown Attenuates Intestinal Injury and Pyroptosis

**Fig. 3:**
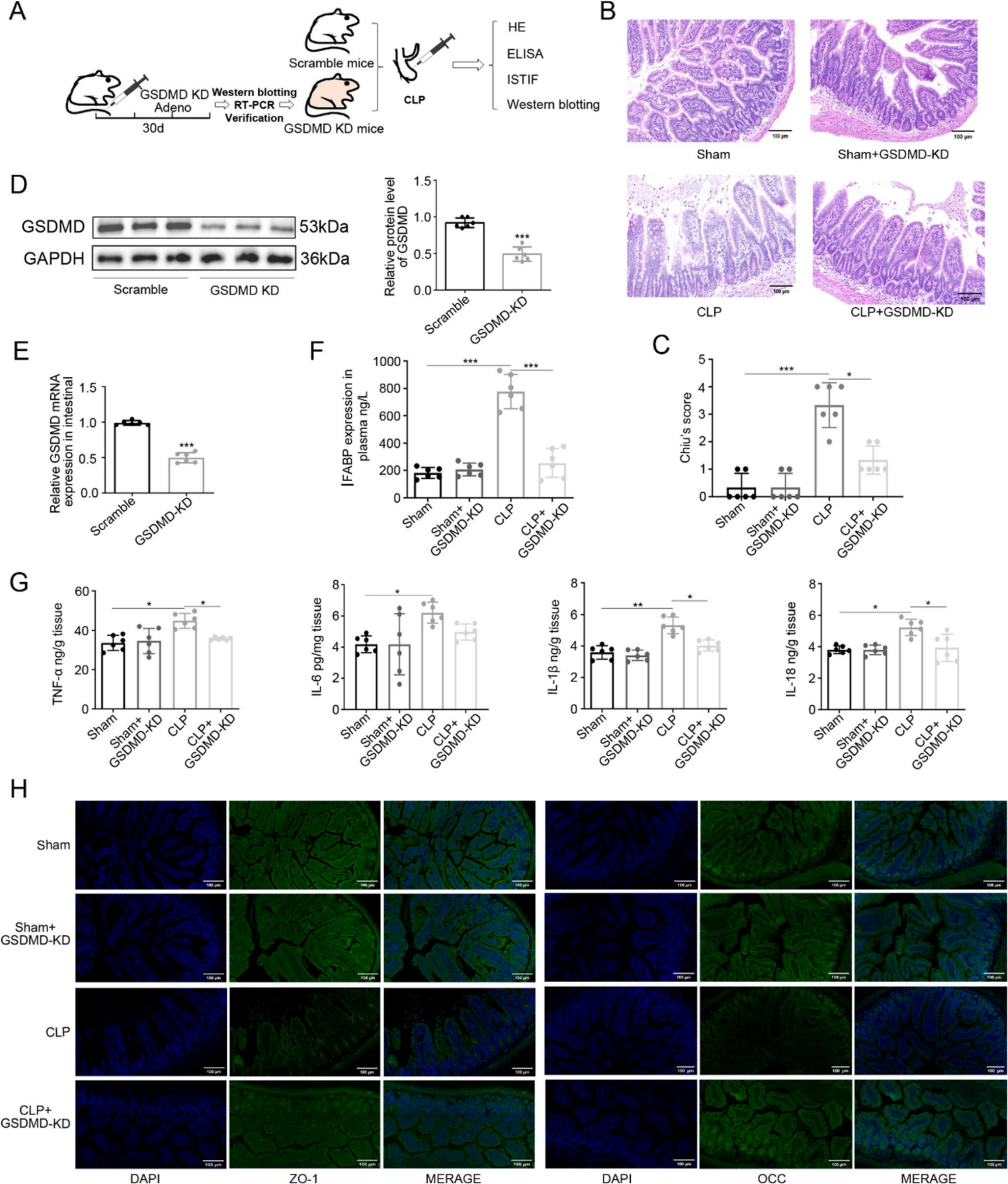
GSDMD knockdown attenuates intestinal injury. **A** Experimental workflow of AAV-shGSDMD delivery. **B**, **C** H&E staining and Chiu's scores showing preserved intestinal architecture in GSDMD-KD CLP mice (*P < 0.05 vs. CLP). **D** GSDMD protein levels in intestinal tissues from Scramble group and GSDMD-KD group (***P < 0.001 vs. Scramble group). **E** >50% GSDMD mRNA reduction in knockdown (KD) mice (***P < 0.001 vs. Scramble group). **F**, **G** Reduced I-FABP (***P < 0.001) and cytokines (TNF-α/IL-1β/IL-18: *P < 0.05 vs. CLP) in GSDMD-KD CLP. **H** Restored ZO-1/occludin expression by immunofluorescence (scale bar: 100 μm). Data: mean ± SD; *n* = 6. Statistical significance was determined by unpaired two tailed Student‘s t test (**D**, **E**), one way ANOVA with Tukey‘s post hoc test (**F**,** G**)

To functionally characterize the role of GSDMD-mediated pyroptosis in sepsis-induced intestinal injury, we generated GSDMD-knockdown (GSDMD-KD) mice through tail vein injection of AAV-shGSDMD (Fig. 3A). Quantitative RT-PCR and western blot confirmed successful knockdown, with both of GSDMD mRNA expression and protein expression were reduced by 50% in intestinal tissues compared to Scramble control (*P* < 0.001; Fig. 3D, E).

Histopathological analysis revealed significant protection in GSDMD-KD mice subjected to CLP. CLP mice exhibited severe intestinal damage, GSDMD-KD CLP mice showed markedly preserved intestinal architecture. Sham group demonstrated no significant differences, confirming the specific protective effect of GSDMD knockdown during sepsis (Fig. 3B, C).

ELISA of intestinal tissue homogenates demonstrated significant protection in GSDMD-KD CLP mice. I-FABP levels were significantly reduced compared to CLP group (*P* < 0.001; Fig. 3F). Pro-inflammatory cytokines including TNF-α, IL-1β and IL-18 showed consistent decreases (*P* < 0.05 for all, CLP+ GSDMD-KD vs. CLP; Fig. 3G), while IL-6 reduction did not reach statistical significance (*P* = 0.075, CLP+ GSDMD-KD vs. CLP; Fig. 3G).

**Fig. 4:**
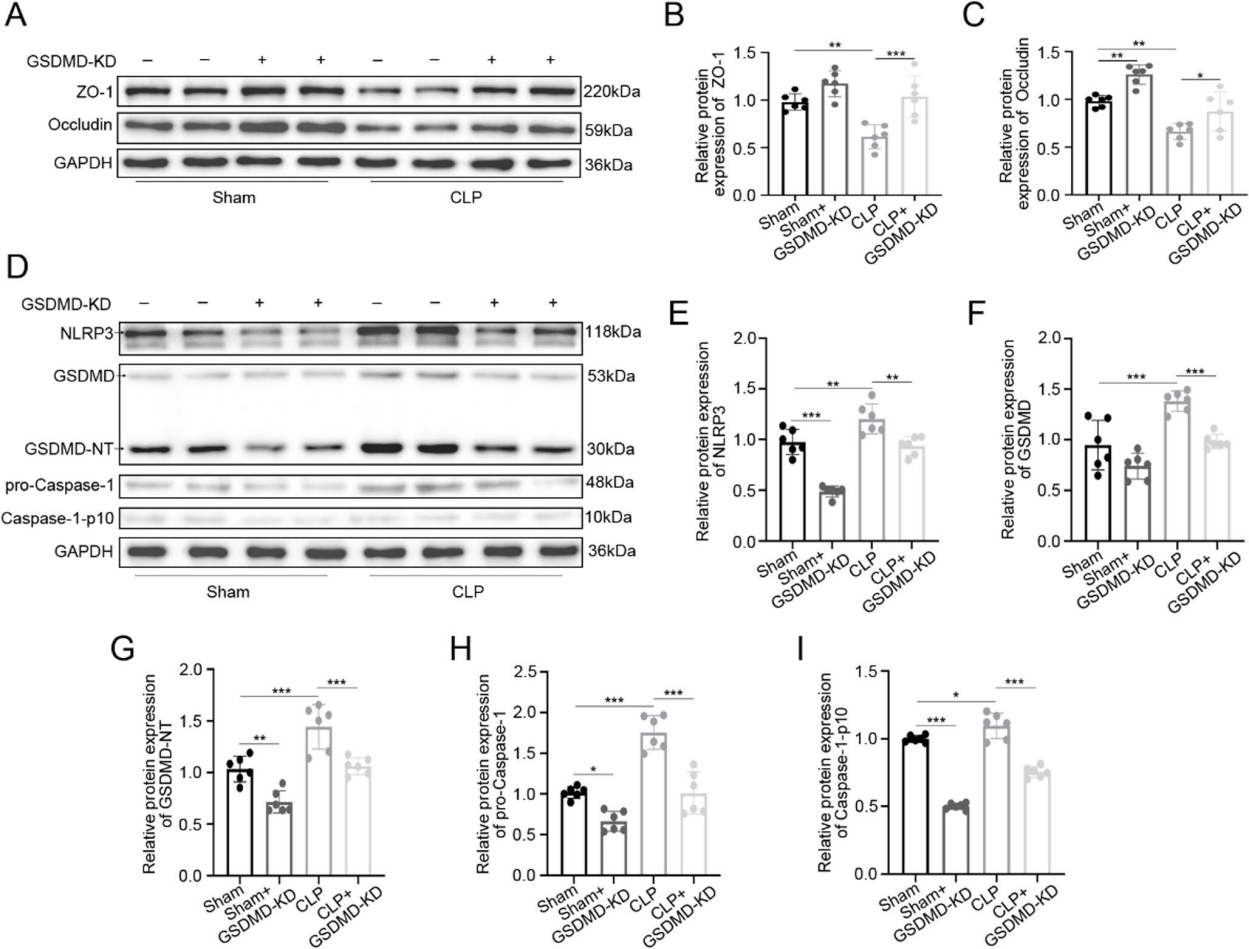
GSDMD knockdown suppresses pyroptotic signaling. **A**-**C** Western blot and quantification of tight junction proteins: ZO-1 (***P < 0.01), occludin (*P < 0.05) in GSDMD-KD CLP vs. CLP. **D**-**I** Reduced pyroptotic markers: NLRP3 (**P < 0.01 vs. CLP), caspase-1-p10 (***P < 0.001 vs. CLP), GSDMD-NT (***P < 0.001 vs. CLP). Data: mean ± SD; *n* = 6. Statistical significance was determined by one way ANOVA with Tukey‘s post hoc test.

Tight junction integrity was assessed through complementary approaches. Immunofluorescence analysis demonstrated restored membrane localization of both ZO-1 and occludin in GSDMD-KD CLP mice compared to the discontinuous staining pattern in CLP mice (Fig. 3H). Western blot quantification confirmed these findings, showing significant preservation of ZO-1 (*P* < 0.01) and occludin (*P* < 0.05) protein expression in GSDMD-KD CLP mice relative to CLP mice (Fig. 4A-C).

At the molecular level, GSDMD knockdown coordinately suppressed pyroptotic pathway components, including NLRP3 (*P* < 0.01), caspase-1-p10 (*P* < 0.05), and GSDMD-NT fragments (*P* < 0.01; Fig. 4D-I). The parallel preservation of tight junction proteins and suppression of pyroptotic effectors in GSDMD-KD mice establishes a mechanistic link between epithelial cell death and barrier dysfunction during sepsis.

### Transcriptomic Profiling Identifies CXCR4 as a Key Regulator in Septic Gut Injury

**Fig. 5:**
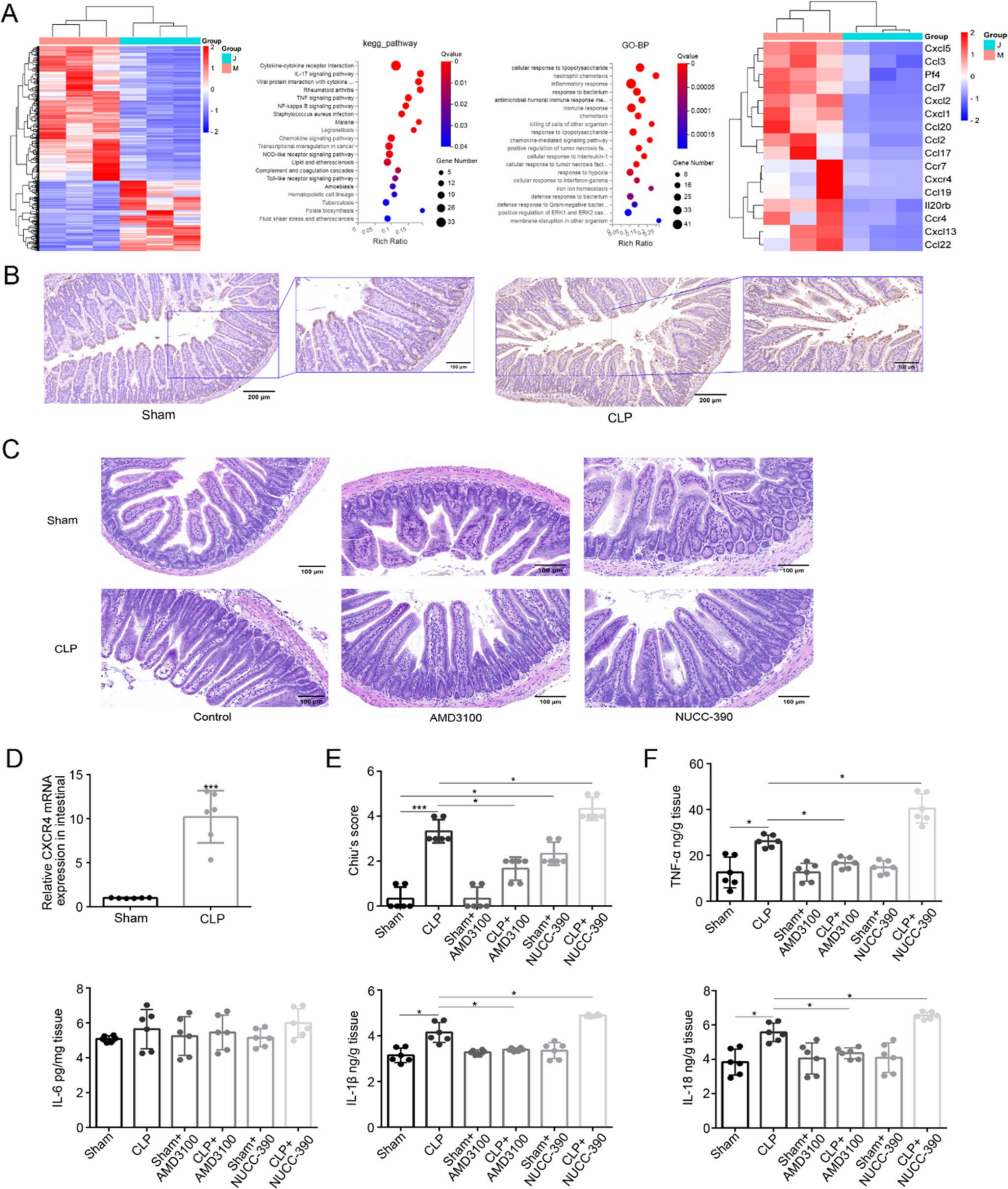
CXCR4 identified as a key regulator of pyroptosis. **A** Heatmap analysis of differentially expressed genes in CLP versus sham intestinal tissues, demonstrating significant upregulation of CXCR4 (log2FC=3.25, Q=0.0101). **B** CXCR4 protein upregulation in CLP intestinal epithelium by IHC (scale bar: 200 μm). **C** H&E staining showing AMD3100 (antagonist) ameliorates injury, while NUCC-390 (agonist) exacerbates it (scale bar: 100 μm). **D** CXCR4 mRNA upregulation in CLP (***P < 0.001 vs. Sham). **E** Chiu's scores: AMD3100 reduces injury (*P < 0.05 vs. CLP), NUCC-390 worsens it (*P < 0.05 vs. CLP). **F** Cytokine modulation by CXCR4 drugs (TNF-α/IL-1β/IL-18: *P < 0.05 vs. CLP). Data: mean ± SD; *n* = 6. Statistical significance was determined by unpaired two tailed Student‘s t test (**D**), one way ANOVA with Tukey‘s post hoc test (**E**, **F**)

To investigate the molecular mechanisms underlying sepsis-induced intestinal injury, we performed RNA sequencing analysis of intestinal tissues from CLP and Sham-operated mice at 12 h post-surgery. Transcriptomic profiling identified CXCR4 as a significantly upregulated gene in CLP mice (log2FC = 3.25, Q value = 0.0101), with notable enrichment in cytokine-cytokine receptor interaction pathways (KEGG pathway analysis, Q value < 0.001) (Fig. 5A).

Validation studies confirmed these findings at both protein and mRNA levels. Immunohistochemical analysis demonstrated marked overexpression of CXCR4 protein specifically within the intestinal epithelium of CLP mice compared to Sham controls (Fig. 5B). Quantitative RT-PCR analysis further confirmed this finding, showing nearly 10-fold increase in CXCR4 mRNA expression in CLP mice versus Sham controls (*P* < 0.001) (Fig. 5D).

The spatial distribution of CXCR4 overexpression correlated with regions exhibiting the most severe epithelial damage, suggesting a potential functional role in barrier disruption. Bioinformatic analysis further identified CXCR4 as a nodal regulator within an inflammatory network containing multiple NF-κB target genes, positioning it as a key candidate mediating pyroptosis-associated intestinal injury during sepsis. Integrating these omics and validation data, we propose a mechanistic model (Fig. 8) wherein epithelial CXCR4 hyperactivation triggers the NF-κB-NLRP3-GSDMD axis, leading to pyroptotic barrier disruption. This model is experimentally tested in subsequent sections.

### Pharmacological Modulation of CXCR4 Alters Intestinal Injury and Pyroptosis

To examine the role of CXCR4 signaling in sepsis-induced intestinal injury, we pharmacologically modulated CXCR4 activity using either the antagonist AMD3100 (5 mg/kg, intravenous) or agonist NUCC-390 (265 mg/kg, intraperitoneal) administered 1 h post-CLP. Histopathological evaluation revealed that CXCR4 inhibition with AMD3100 significantly attenuated intestinal damage (*P* < 0.05 versus untreated CLP), while CXCR4 activation with NUCC-390 exacerbated injury (*P* < 0.05 versus CLP alone) (Fig. 5C, E). Notably, NUCC-390 administration in Sham-operated mice induced mild but significant intestinal damage (*P* < 0.05 versus Sham), suggesting a physiological role for CXCR4 in mucosal homeostasis.

Analysis of inflammatory mediators demonstrated that AMD3100 treatment significantly reduced CLP-induced elevations in TNF-α, IL-1β, and IL-18 (*P* < 0.05 for all), whereas NUCC-390 further potentiated their expression (*P* < 0.05 vs. CLP). IL-6 levels showed no significant modulation by either treatment (*P* > 0.05; Fig. 5F). These coordinated changes in responsive cytokines indicate CXCR4 signaling selectively regulates specific inflammatory pathways during sepsis.

**Fig. 6:**
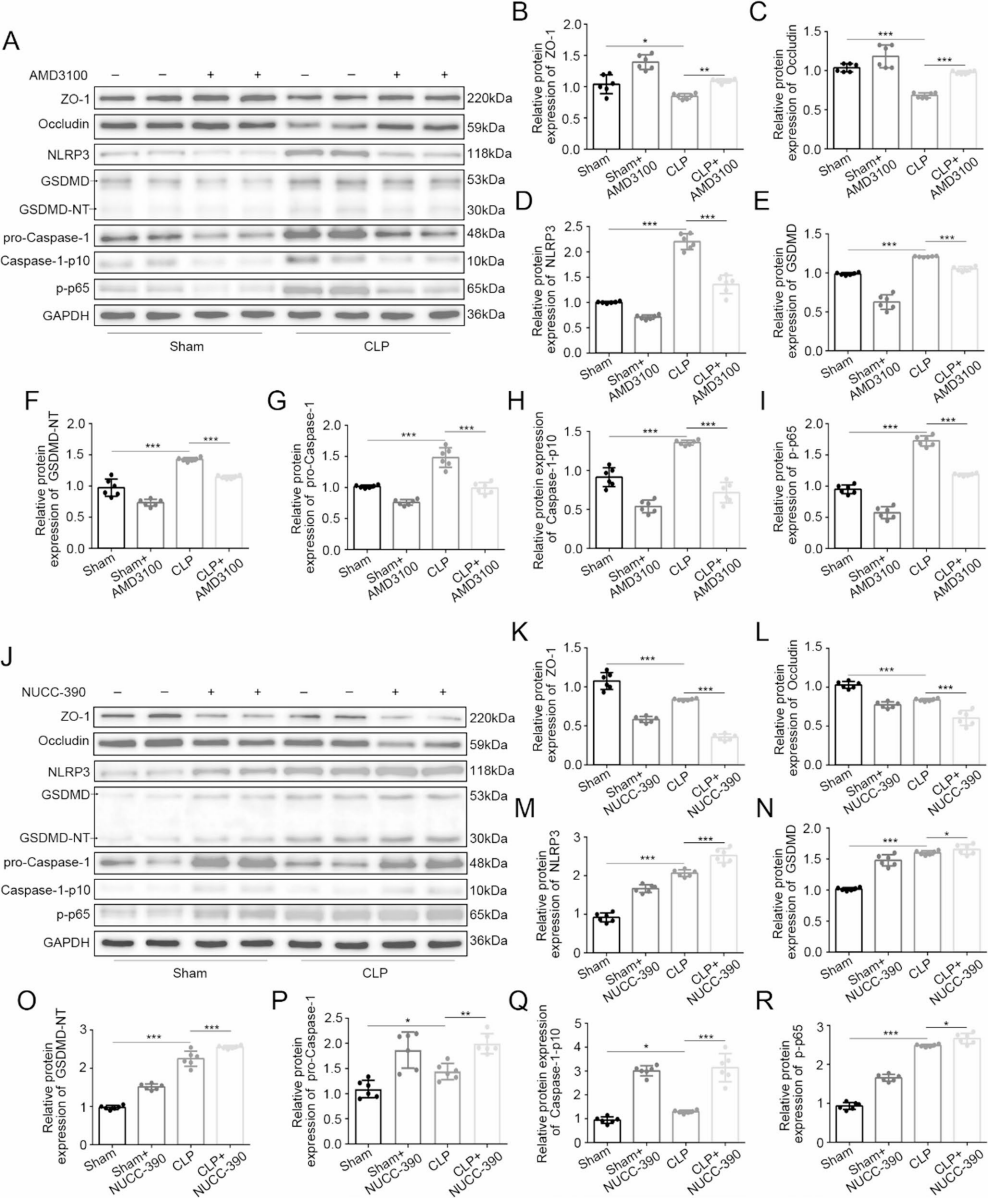
CXCR4 modulates pyroptosis through NF-κB/NLRP3 axis. **A**, **B**, **C**, **J**, **K**, **L** Western blot and quantification:Tight junction proteins: ZO-1/occludin reduced by NUCC-390 (***P < 0.001 vs. CLP), rescued by AMD3100(**P < 0.01, ***P < 0.001 vs. CLP). **A**, **D**-**I**, **J**, **M**-**R** Pyroptotic markers: NLRP3, GSDMD-NT and caspase-1-p10 reduced by AMD3100 (***P < 0.001 vs. CLP); upregulated by NUCC-390 (*P < 0.05, **P < 0.01, ***P < 0.001 vs. CLP). Phospho-NF-κB p65 (Ser536) increased by NUCC-390 (*P < 0.05 vs. CLP), suppressed by AMD3100 (***P < 0.001 vs. CLP). Data: mean ± SD; *n* = 6. Statistical significance was determined by one way ANOVA with Tukey‘s post hoc test.

At the tight junction level, AMD3100 preserved ZO-1 and occludin expression (*P* < 0.01 for ZO-1, *P* < 0.001 for occluding, versus CLP), while NUCC-390 exacerbated their downregulation (*P* < 0.001) (Fig. 6A, B, C, J, K, L). Intriguingly, NUCC-390 alone induced a noticeable reduction of these junctional proteins even in Sham mice, suggesting a pathogenic potential of CXCR4 hyperactivation independent of septic insult. These morphological and biochemical improvements correlated with suppression of the pyroptotic cascade. AMD3100 significantly reduced expression of NLRP3, GSDMD-N, and caspase-1-p10 (*P* < 0.001, versus CLP), whereas NUCC-390 enhanced their induction (*P* < 0.05, *P* < 0.01, *P* < 0.001, versus CLP) (Fig. 6A, D-H, J, M-Q). Notably, administration of NUCC-390 alone in Sham mice was sufficient to significantly upregulate these pyroptotic markers and activate the pathway, confirming that CXCR4 agonism can directly trigger inflammasome signaling and pyroptosis even in the absence of septic injury. The consistent responses observed in both CLP and Sham groups demonstrate that CXCR4 signaling intrinsically regulates the NLRP3-GSDMD axis, independent of sepsis-induced pathway activation.

**Fig. 7:**
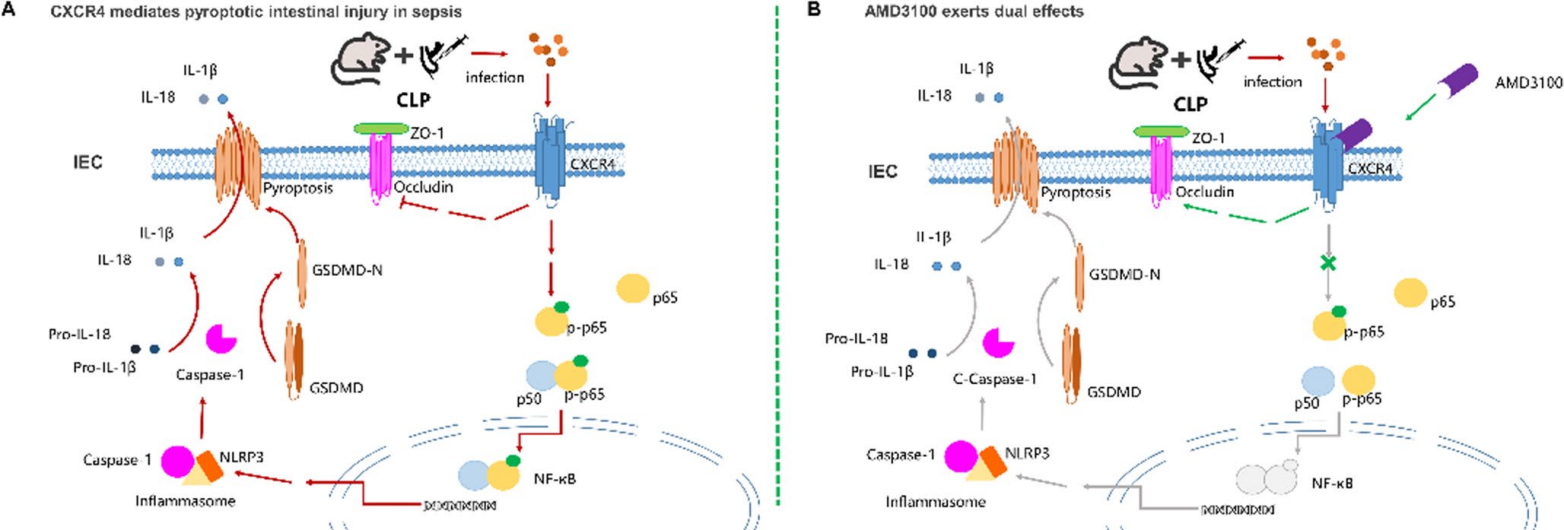
CXCR4 drives intestinal epithelial pyroptosis via the NLRP3-GSDMD axis in sepsis. Sepsis pathogens activate CXCR4, driving NF-κB-dependent NLRP3 inflammasome assembly and caspase-1-mediated GSDMD cleavage. This results in pyroptotic epithelial cell death and intestinal barrier disruption. Pharmacological inhibition of CXCR4 by AMD3100 or genetic ablation of GSDMD blocks this cascade

Mechanistic investigation of the NF-κB pathway revealed that AMD3100 inhibited p65 phosphorylation at Ser536 (*P* < 0.001, versus CLP), while NUCC-390 enhanced phosphorylation (*P* < 0.05 versus CLP) (Fig. 6A, I, J, R). Similarly, NUCC-390 also promoted significant p65 phosphorylation in Sham controls, reinforcing its role as a primary upstream activator of NF-κB. These results establish CXCR4 as an upstream regulator of NF-κB-dependent NLRP3 inflammasome activation and subsequent GSDMD-mediated pyroptosis in septic intestinal injury, providing a mechanistic basis for the observed protection with CXCR4 inhibition (Fig. 7).

### CXCR4-GSDMD Crosstalk via NF-κB/NLRP3 Axis

**Fig. 8:**
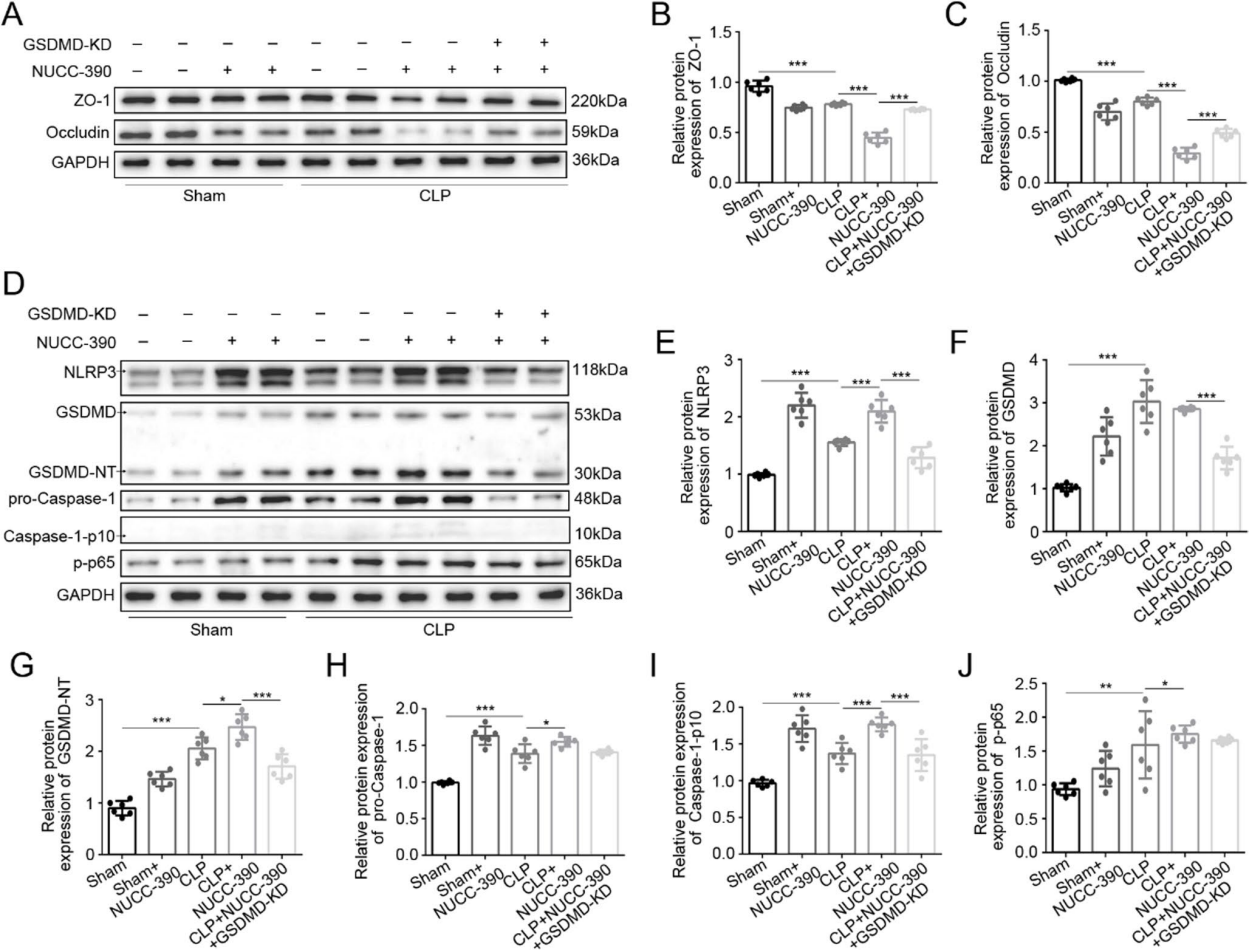
GSDMD dependency in CXCR4-mediated barrier disruption. **A**-**C** NUCC-390-induced ZO-1/occludin downregulation (***P < 0.001 vs. CLP) is rescued in GSDMD-KD mice (***P < 0.01 vs. CLP+NUCC-390). **D**-**J** GSDMD-KD blunts NUCC-390-induced pyroptotic markers (NLRP3: ***P < 0.01; GSDMD-NT: ***P < 0.01; caspase-1-p10: ***P < 0.01 vs CLP+NUCC-390) but not p65 phosphorylation (*P* > 0.05). Data: mean ± SD; *n* = 6. Statistical significance was determined by one way ANOVA with Tukey‘s post hoc test

To mechanistically dissect the regulatory relationship between CXCR4 signaling and GSDMD-mediated pyroptosis, we employed a genetic approach using GSDMD-KD mice. Pharmacological activation of CXCR4 with NUCC-390 in CLP mice markedly exacerbated intestinal barrier dysfunction, as demonstrated by further reduction in tight junction proteins ZO-1 and occludin (*P* < 0.001, versus CLP alone). However, this detrimental effect was substantially attenuated in GSDMD-KD mice (*P* < 0.001 versus CLP+NUCC-390), indicating GSDMD dependency for CXCR4-mediated barrier disruption (Fig. 8A-C).

Molecular characterization revealed that while NUCC-390 treatment significantly enhanced expression of pyroptotic components (NLRP3, caspase-1-p10, and GSDMD-NT fragments) in CLP mice (*P* < 0.05, *P* < 0.001, versus CLP), these effects were significantly blunted in GSDMD-KD CLP mice (*P* < 0.001, versus CLP+NUCC-390) (Fig. 8D-I). Crucially, the phosphorylation of NF-κB p65 induced by NUCC-390 was not attenuated by GSDMD knockdown (*P* > 0.05 versus CLP+NUCC-390), definitively placing NF-κB activation upstream of GSDMD cleavage (Fig. 8D, J).

These findings establish a definitive signaling hierarchy wherein CXCR4 activation drives NF-κB-dependent upregulation of the NLRP3 inflammasome, leading to caspase-1-mediated GSDMD cleavage and subsequent pyroptotic cell death. The spatial correlation between CXCR4 overexpression and epithelial damage regions, combined with the differential rescue of downstream signaling events in GSDMD-KD mice, provides compelling evidence that CXCR4 orchestrates intestinal barrier dysfunction through this NF-κB/NLRP3/GSDMD axis during sepsis (Fig. 7).

### NF-κB Inhibition Rescues CXCR4-Mediated Barrier Dysfunction and Pyroptosis

**Fig. 9:**
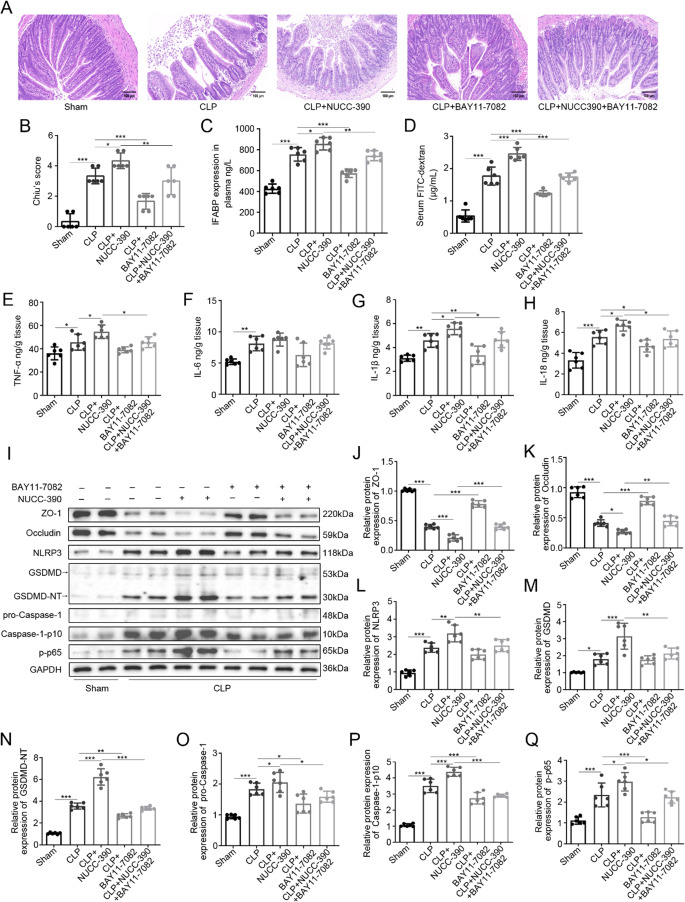
NF κB inhibition rescues CXCR4 mediated barrier dysfunction and pyroptosis. **A**, **B** H&E staining and Chiu‘s score showing BAY 11 7082 attenuates CLP induced injury (***P < 0.001 vs. CLP) and reverses NUCC 390 induced exacerbation (**P < 0.01 vs. CLP+NUCC 390). **C, D** I FABP levels and FITC dextran permeability assay. BAY 11 7082 reduces injury marker and barrier dysfunction in CLP (***P < 0.001 vs. CLP) and CLP+NUCC 390 groups (**P < 0.01, ***P < 0.001 vs. CLP+NUCC 390). **E**, **H** Inflammatory cytokines. NUCC 390 potentiates CLP induced TNF α, IL 1β, IL 18 (*P < 0.05 vs. CLP), effects reversed by BAY 11 7082 (*P < 0.05 vs. CLP+NUCC 390). IL 6 is elevated in CLP (**P < 0.01 vs. Sham) but unchanged by treatments. (I Q) Western blot analysis of tight junction and pyroptosis pathway proteins. BAY 11 7082 restores ZO 1/occludin, suppresses p p65, NLRP3 (in NUCC 390 group), caspase 1 p10, and GSDMD NT (*P < 0.05, **P < 0.01, ***P < 0.001 vs. indicated groups). Data: mean ± SD (dot plots); *n* = 6. Statistics: one way ANOVA with Tukey‘s post hoc test

To definitively establish NF-κB as a critical downstream node of CXCR4 signaling, we employed the NF-κB inhibitor BAY 11-7082 in a rescue experiment. Consistent with our previous findings, CLP induced significant intestinal damage compared to Sham controls (*P* < 0.001). This damage was significantly attenuated by BAY 11-7082 treatment (*P* < 0.001 versus CLP). Furthermore, the exacerbation of mucosal injury by the CXCR4 agonist NUCC-390 (*P* < 0.05 versus CLP) was markedly reversed by co-administration of BAY 11-7082 (*P* < 0.01 versus CLP+NUCC-390) (Fig. 9A, B).

Assessment of intestinal barrier function using the FITC-dextran assay demonstrated a severe increase in permeability in CLP mice (*P* < 0.001 versus Sham), which was further aggravated by NUCC-390 (*P* < 0.001 versus CLP). Critically, inhibition of NF-κB with BAY 11-7082 not only reduced the hyperpermeability caused by CLP alone (*P* < 0.001 versus CLP) but also abolished the detrimental effect of NUCC-390 (*P* < 0.001 versus CLP+NUCC-390) (Fig. 8D). Consistently, levels of the epithelial injury marker I-FABP, elevated in CLP mice (*P* < 0.001 versus Sham) and further increased by NUCC-390 (*P* < 0.05 versus CLP), were significantly attenuated by BAY 11-7082 co-treatment (*P* < 0.01, *P* < 0.001 versus respective groups without inhibitor) (Fig. 9C).

Molecular analysis of the inflammatory cascade showed that CLP-induced elevations in TNF-α, IL-1β, and IL-18 (*P* < 0.05, *P* < 0.01, *P* < 0.001 versus Sham) were further potentiated by NUCC-390 (*P* < 0.05 versus CLP for all). Co-treatment with BAY 11-7082 significantly suppressed the levels of these cytokines in mice that received NUCC-390 (*P* < 0.05 versus CLP+NUCC-390) (Fig. 9E, G, H). BAY 11-7082 alone reduced TNF-α, IL-1β and IL-18 in CLP mice, although this reduction reached statistical significance for IL-1β and IL-18 (*P* < 0.05, *P* < 0.01 versus CLP) but not for TNF-α (*P* > 0.05) (Fig. 9E, G, H). IL-6 levels were elevated in CLP (*P* < 0.01 versus Sham) but were not significantly modulated by either NUCC-390 or BAY 11-7082 (Fig. 9 F).

At the level of barrier structure, the degradation of tight junction proteins ZO-1 and occludin observed in CLP mice (*P* < 0.001 versus Sham) was exacerbated by NUCC-390 (*P* < 0.001, *P* < 0.05 versus CLP). BAY 11-7082 treatment significantly restored the expression of both ZO-1 and occludin in CLP mice (*P* < 0.001 versus CLP) and, most importantly, reversed their NUCC-390-induced downregulation (*P* < 0.001, *P* < 0.01 versus CLP+NUCC-390) (Fig. 9 I-K).

Mechanistic interrogation of the signaling axis revealed that BAY 11-7082 effectively suppressed the phosphorylation of NF-κB p65 (Ser536) induced by both CLP and NUCC-390 (*P* < 0.001, *P* < 0.05 versus respective groups without inhibitor) (Fig. 9 I, Q). Consequently, it downregulated key executors of pyroptosis: the cleavage of GSDMD (GSDMD-NT) and pro-caspase-1 (caspase-1-p10) was significantly reduced in both CLP and CLP+NUCC-390 groups (*P* < 0.01 to *P* < 0.001 versus respective groups without inhibitor) (Fig. 9I, N, P). Furthermore, the upregulation of NLRP3 protein by NUCC-390 was inhibited by BAY 11-7082 (*P* < 0.01 versus CLP+NUCC-390).

These results collectively demonstrate that NF-κB activation is an indispensable mechanistic link, functionally coupling CXCR4 hyperactivation to NLRP3 inflammasome-dependent GSDMD cleavage, pyroptosis, and ultimate intestinal barrier failure during sepsis.

## Discussion

Our study delineates a previously unrecognized pathogenic axis wherein CXCR4 orchestrates NLRP3/GSDMD-mediated pyroptosis to drive septic intestinal barrier failure. Crucially, we demonstrate that pharmacological inhibition of CXCR4 with AMD3100 concurrently suppresses epithelial pyroptosis, attenuates inflammation, and preserves barrier integrity—revealing a promising preclinical therapeutic strategy with repurposing potential. By integrating transcriptomics, genetic knockdown, and targeted pharmacology, we establish that CXCR4 activation promotes NF-κB p65 phosphorylation (Ser536), triggering NLRP3-dependent GSDMD cleavage and pyroptotic epithelial death. Most importantly, genetic ablation of GSDMD rescues barrier damage without affecting upstream NF-κB activation, providing definitive genetic evidence for the signaling hierarchy. To further cement this hierarchy and establish causality, we performed a pivotal rescue experiment using an NF-κB inhibitor. We found that pharmacological inhibition of NF-κB not only ameliorated CLP-induced injury but, most critically, completely abrogated the detrimental effects of CXCR4 agonism on barrier integrity, inflammation, and the entire pyroptotic cascade. This provides direct functional evidence that NF-κB is an indispensable and obligate signaling node downstream of CXCR4 and upstream of NLRP3/GSDMD activation in the septic gut (Fig. 7).

Accumulating evidence underscores the pivotal role of NLRP3/GSDMD-mediated pyroptosis as a central executioner in sepsis-induced intestinal injury, making it a critical therapeutic target. This concept is strongly supported by recent mechanistic and interventional studies: for instance, UCP2 deficiency was shown to exacerbate intestinal damage through enhanced NLRP3-mediated pyroptosis [23], whereas pharmacological inhibition of PFKFB3 preserved barrier integrity by suppressing the NLRP3/GSDMD pathway [24]. Additionally, natural compounds such as Nano Acacetin have demonstrated efficacy in mitigating mucosal injury by improving mitochondrial function and regulating TRX1 to inhibit NLRP3-dependent pyroptosis [17], and Qi Huang Fang was reported to improve intestinal barrier function via modulation of the same pathway [25]. MicroRNA-based mechanisms also contribute to this process, as evidenced by miR-155 which promotes epithelial damage through SIRT1/NF-κB-mediated activation [15], and miR-874-5p which targets VDR to reduce pyroptosis [16]. Even gaseous mediators like carbon monoxide have been found to inhibit expression of pyroptosis-related proteins in septic intestines [26].

Our results align with and extend these findings. In a CLP-induced septic model, we confirmed the activation of pyroptotic events in intestinal tissues, with immunofluorescence co-localization demonstrating GSDMD activation specifically within CK19⁺ enterocytes—consistent with histopathological features of barrier dysfunction [27]. Using GSDMD-knockdown mice, we observed significant attenuation of intestinal injury, preservation of tight junction proteins (ZO-1 and occludin), and reduction in pro-inflammatory cytokines. At the molecular level, GSDMD knockdown suppressed pyroptosis-executing proteins including NLRP3, cleaved caspase-1, and GSDMD-N terminal fragments, underscoring the crucial role of GSDMD-dependent pyroptosis in septic intestinal pathology.

While the aforementioned studies reveal multiple upstream modulators of pyroptosis, they predominantly target intracellular components—such as miRNAs, metabolic enzymes, or mitochondrial regulators—which pose challenges for clinical translation due to delivery limitations and off-target potential. Our work fundamentally advances this field by identifying CXCR4, a druggable G protein-coupled receptor located on the cell surface, as a key upstream regulator orchestrating the NF-κB/NLRP3/GSDMD axis. Unlike intracellular agents, CXCR4 is readily targetable; using the FDA-approved inhibitor AMD3100, we demonstrated that blockade of CXCR4 abrogates the entire pyroptotic cascade. This was further validated by our rescue experiment, where inhibition of the downstream effector NF-κB phenocopied the protective effects of CXCR4 blockade, confirming the linearity and druggability of this pathway. This not only provides a mechanistically clear and therapeutically actionable pathway but also highlights its clinical relevance for drug repurposing in sepsis management.

CXCR4, a 352-amino acid rhodopsin-like GPCR, is ubiquitously expressed and plays context-dependent roles in both physiological homeostasis and pathological processes [28]. While extensively studied in oncology for its roles in tumor metastasis and angiogenesis [29–31], emerging evidence implicates CXCR4 in regulating inflammatory cell death across various disease models. For instance, CXCR4 signaling promotes NLRP3-mediated pyroptosis in subarachnoid hemorrhage-induced brain injury [19], sciatic nerve injury [32], and intervertebral disc degeneration [20], suggesting a conserved mechanism in inflammatory tissue damage.

Our findings extend this paradigm to sepsis-induced intestinal injury. Transcriptomic profiling identified CXCR4 as one of the most significantly upregulated genes in septic intestines, a finding validated at both mRNA and protein levels via qPCR and immunohistochemistry. Notably, CXCR4 overexpression was most pronounced in regions exhibiting severe epithelial disruption and inflammatory infiltrate, suggesting a spatial correlation between CXCR4 expression and tissue damage. Functional studies using pharmacological modulators further underscore its central role: inhibition with AMD3100 markedly attenuated intestinal injury, while agonism with NUCC-390 exacerbated epithelial barrier failure and pyroptosis. Intriguingly, NUCC-390 alone induced measurable barrier disruption even in Sham group mice (Fig. 6C, D), implying that CXCR4 activation is sufficient to provoke pathology-independent barrier dysfunction. This pathological role stands in stark contrast to its well-established physiological function in maintaining intestinal epithelial integrity, where the CXCL12/CXCR4 axis is critical for regulating epithelial cell migration, epithelial restitution, and promotion of intestinal barrier integrity [33, 34]. The stark contrast between its homeostatic and pathological functions supports a model of bidirectional regulability (Fig. 9). Under physiological conditions, tonic CXCR4 signaling appears to contribute to mucosal homeostasis; however, during sepsis, its hyperactivation drives NF-κB-dependent pyroptosis, disrupting barrier integrity. This Janus-faced character positions CXCR4 as a dynamically tunable target, offering therapeutic leverage to restore homeostasis in hyperinflammatory states like sepsis.

Notably, our bioinformatic analysis of septic intestinal transcriptomes revealed significant enrichment in cytokine-cytokine receptor interactions and NF-κB signaling pathways, positioning CXCR4 within a broader inflammatory network. This computational insight guided our subsequent mechanistic investigations, which definitively established CXCR4 as an upstream regulator of the NF-κB/NLRP3/GSDMD axis. Specifically, we demonstrated that CXCR4 activation enhances NF-κB p65 phosphorylation at Ser536, thereby propelling NLRP3 inflammasome assembly, caspase-1 activation, and GSDMD cleavage. Crucially, genetic ablation of GSDMD abolished CXCR4-driven barrier injury and pyroptosis without attenuating NF-κB activation, underscoring GSDMD’s role as the indispensable terminal executor downstream of CXCR4/NF-κB signaling. Thus, our integrated bioinformatic and experimental approach delineates a linear CXCR4–NF-κB–NLRP3–GSDMD signaling hierarchy, providing both a novel mechanistic framework for sepsis-induced organ dysfunction and a compelling rationale for therapeutic targeting of this pathway.

Current clinical management of septic intestinal injury remains predominantly supportive, relying on antibiotics, hemodynamic stabilization, and enteral nutrition to control infection and maintain organ perfusion. However, a critical therapeutic gap persists: the absence of agents specifically designed to protect and restore the intestinal barrier [35]. Our discovery that pharmacological inhibition of CXCR4 simultaneously suppresses pyroptosis, attenuates inflammation, and preserves barrier integrity presents a compelling strategy to fill this unmet need. Importantly, the translational appeal of this approach is greatly amplified by the clinical availability of AMD3100 (plerixafor), an FDA-approved CXCR4 antagonist. While CXCR4-targeting agents have been explored in oncology for their ability to induce pyroptosis [36–38], our work paradoxically demonstrates that inhibiting the same receptor in sepsis exerts potent protective effects, highlighting the critical importance of disease context. The prospect of repurposing AMD3100 offers a tangible path toward rapid clinical translation, potentially in combination with adjunctive anti-inflammatory therapies to achieve synergistic efficacy at reduced doses.

The clinical relevance of targeting CXCR4 in sepsis is further underscored by human studies. Elevated CXCR4 expression has been documented in circulating leukocytes of septic patients compared to non-septic critically ill controls [39]. Our findings extend these observations beyond mere association by providing a mechanistic link between CXCR4 hyperactivation and intestinal barrier failure via GSDMD-mediated pyroptosis. This suggests that modulating the CXCR4/NF-κB/NLRP3/GSDMD axis, particularly through epithelium-targeted approaches, could yield significant barrier-protective benefits without broadly disrupting the physiological functions of CXCR4 in other organ systems.

Despite the compelling evidence presented herein, our study has several limitations that also delineate clear directions for future research. First, although we have established the CXCR4–NF-κB–NLRP3–GSDMD axis in vivo, the precise molecular mechanism that couples CXCR4 activation to NF-κB phosphorylation in intestinal epithelial cells remains incompletely defined. Future work employing intestinal epithelial-specific knockout models or reductionist in vitro systems will be necessary to dissect whether this coupling involves direct receptor signalling or requires intermediate adaptor proteins. Second, as a preclinical study, we have not yet validated the activation of this pathway in clinical samples from septic patients. Subsequent clinical investigations are needed to measure the expression of CXCR4 and downstream pathway components in patients and to correlate their levels with the severity of intestinal injury and clinical outcomes—a step crucial for identifying potential subgroups that might benefit from CXCR4-targeted therapy. Third, our findings are confined to the early hyperinflammatory phase of sepsis (12 h post-CLP). Given the biphasic nature of sepsis, the dynamics of this axis during the later immunosuppressive and recovery phases remain entirely unexplored and warrant dedicated investigation. Finally, although our genetic and pharmacological rescue data support a mechanism-specific action of NUCC-390, a more comprehensive pharmacological characterisation (e.g., detailed dose-response relationships) would further solidify its utility as a research tool. To advance the translational potential of these findings, dedicated follow-up studies are required to optimise AMD3100 dosing regimens for sepsis, to identify predictive biomarkers, and to evaluate its efficacy—either alone or in rational combinations—in higher-order preclinical models that more faithfully recapitulate human septic pathophysiology.

## Conclusions

In conclusion, our study delineates the CXCR4/NF-κB/NLRP3/GSDMD axis as a critical and druggable signaling pathway driving pyroptosis and barrier disruption in septic intestinal injury. By identifying the cell-surface receptor CXCR4 as a master upstream regulator of this cascade, we provide a novel mechanistic framework for sepsis-induced organ dysfunction and highlight a promising, therapeutically actionable target. The efficacy of the FDA-approved agent AMD3100 in simultaneously suppressing inflammation and preserving epithelial integrity supports its potential for repurposing as a barrier-protective therapy. Moving forward, prioritizing translational studies—including dose optimization, biomarker stratification, and combination regimens—will be essential to bridge these compelling preclinical findings to clinical applications, ultimately offering a targeted approach to mitigate intestinal failure and improve outcomes in sepsis.

## Supplementary Information

Below is the link to the electronic supplementary material.

Supplementary Material 1

## Data Availability

The datasets supporting the conclusions of this article are available from the corresponding author on reasonable request. The sequencing data has been uploaded to the NCBI BioProject and the submission number is PRJNA1279816.
