# Supplementary material for: Pharmacological Targeting of CXCR4 Attenuates Sepsis-Induced Intestinal Injury by Suppressing NLRP3/GSDMD-Mediated Pyroptosis
Source: Inflammation. 2026 Apr 2;49(1):140. doi: 10.1007/s10753-026-02494-7 (PMC13171779; doi:10.1007/s10753-026-02494-7)

Fig 2

NLRP3

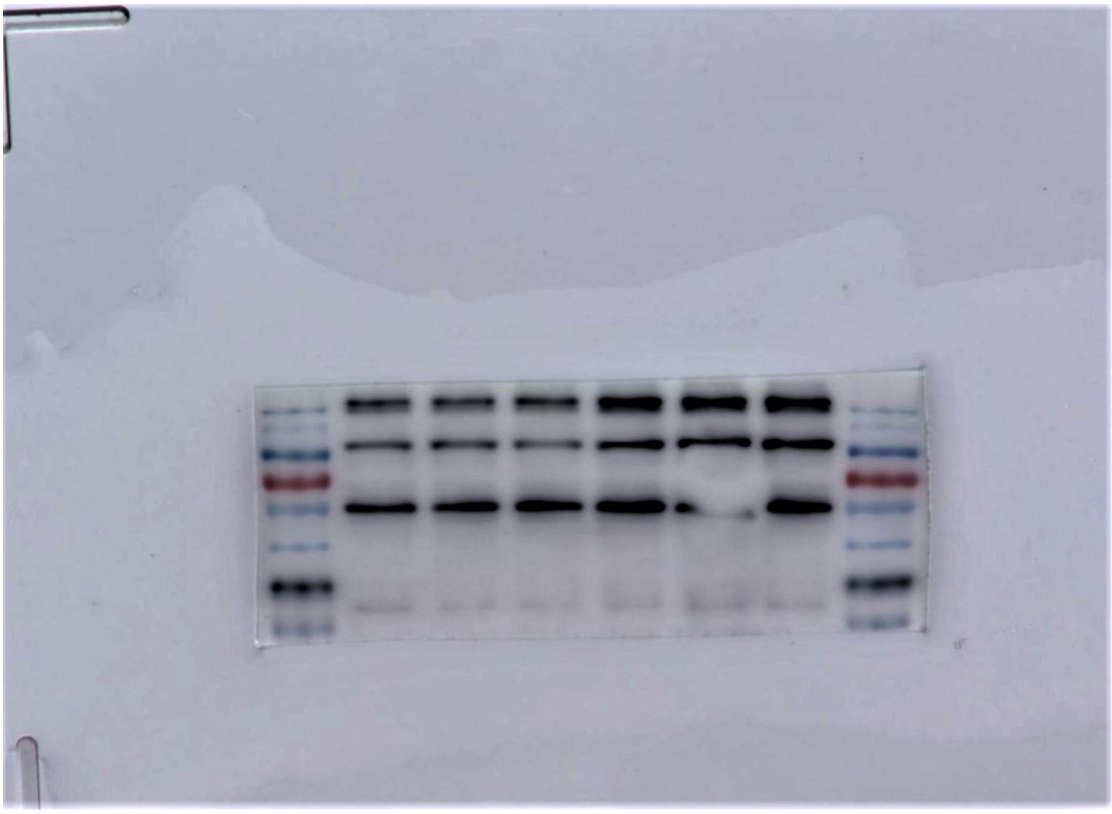

GSDMD/GSDMD-N

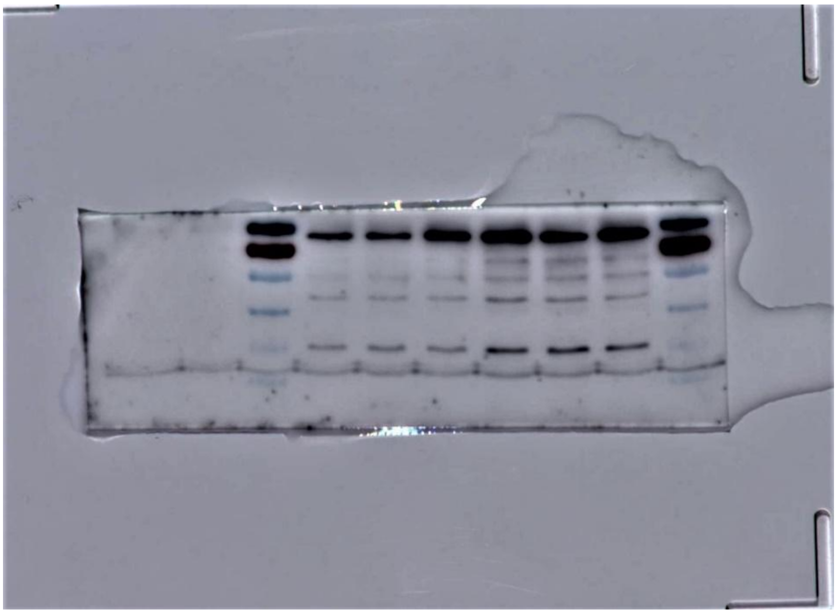

Caspase-1/ Caspase-1-p10

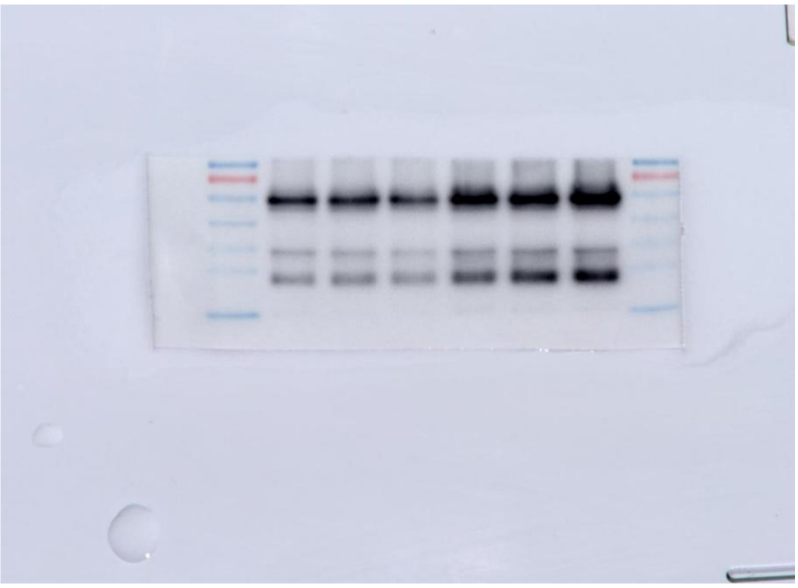

GAPDH

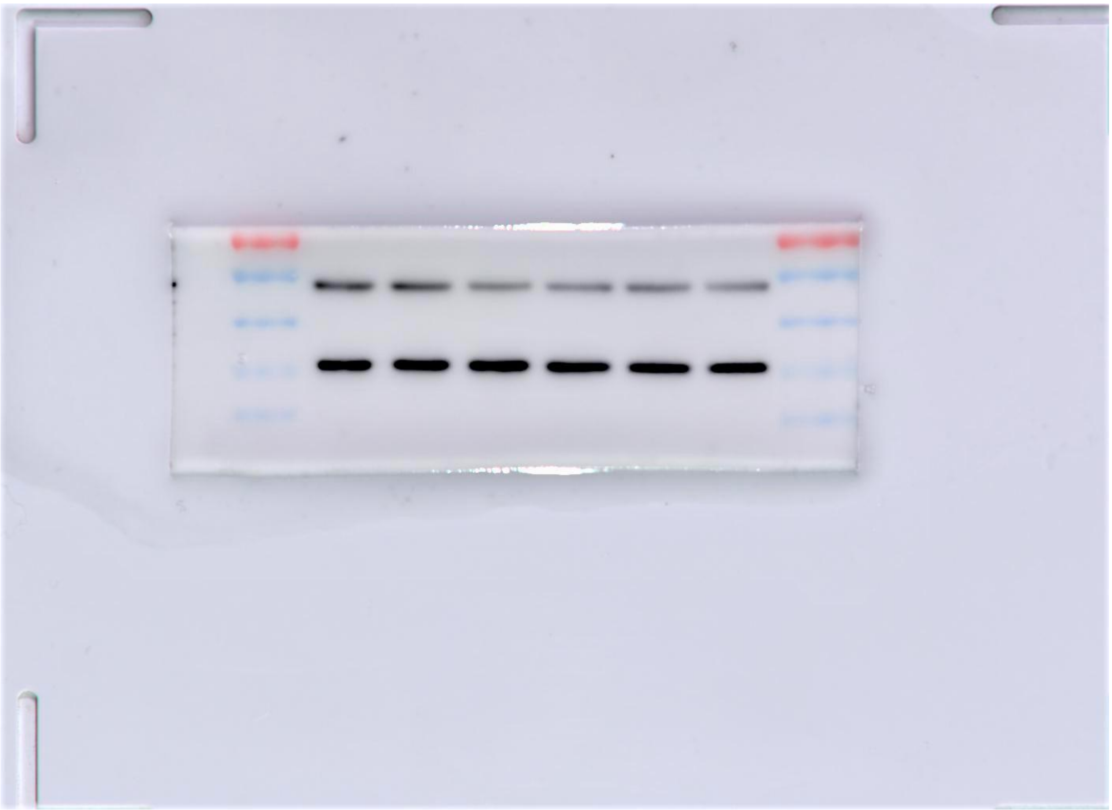

Fig 3

GSDMD

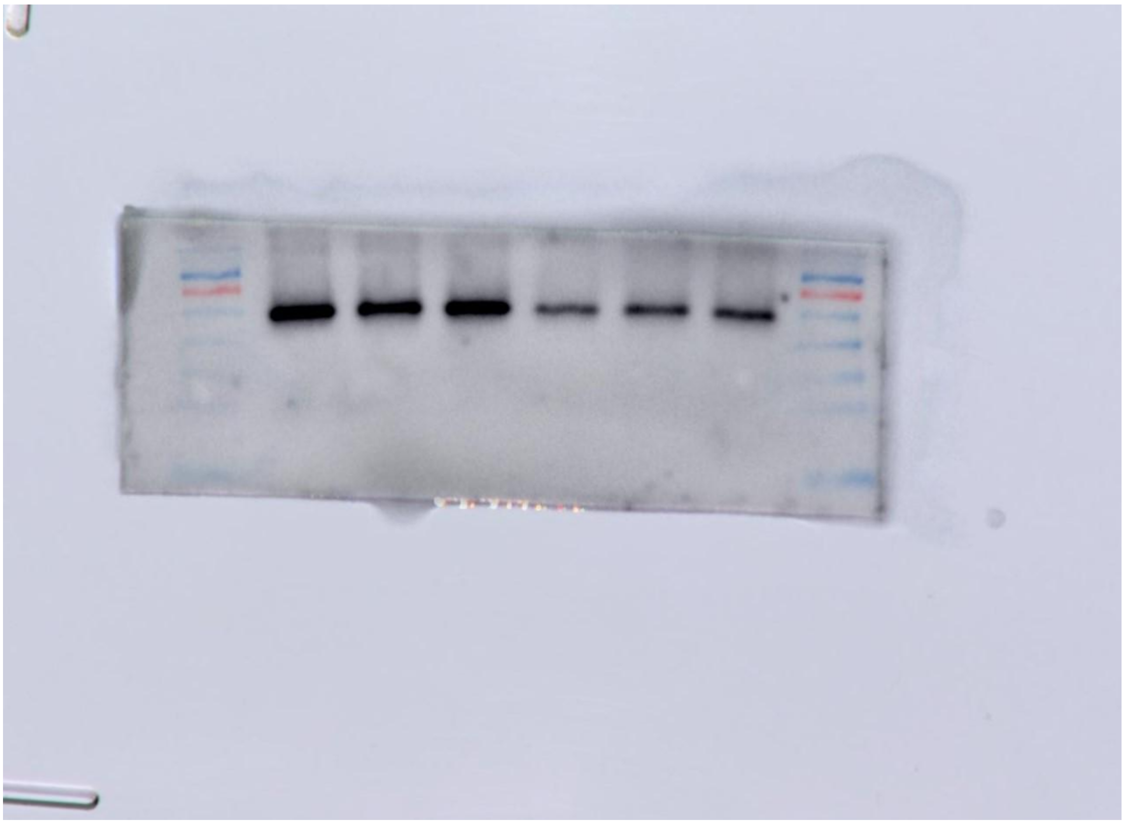

GAPDH

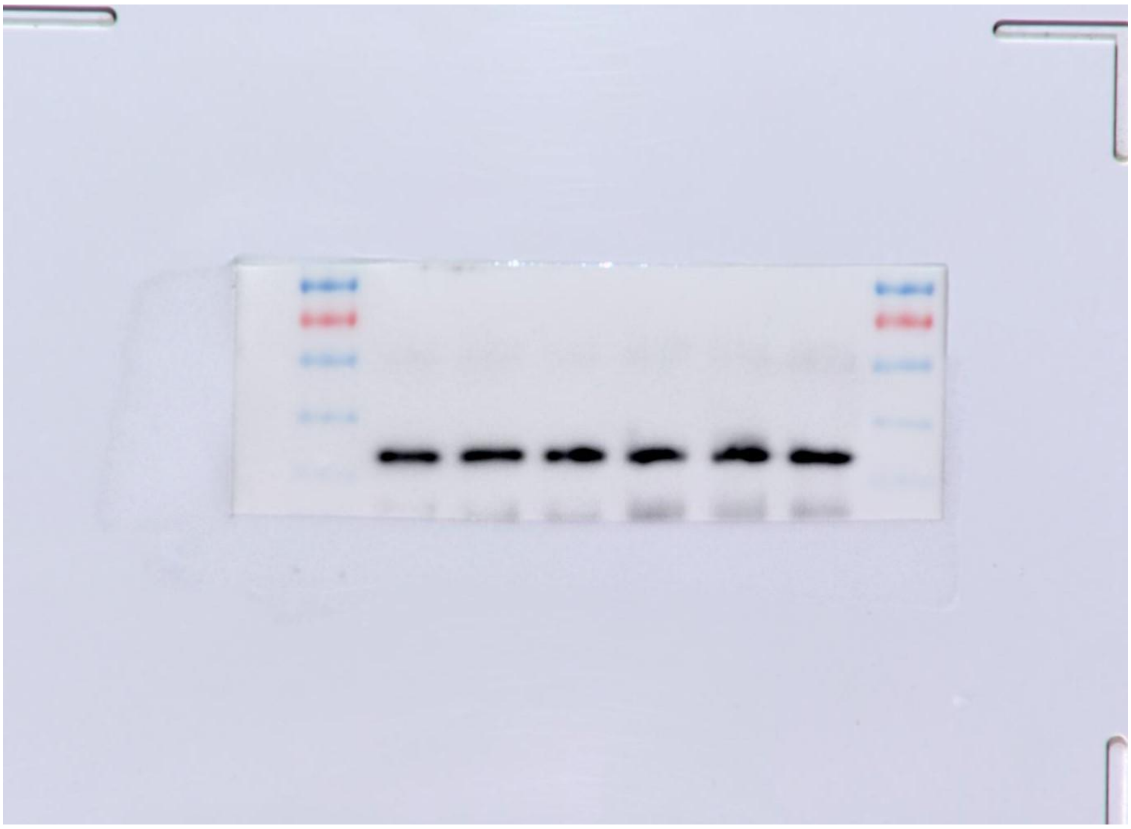

Fig 4

Zo-1

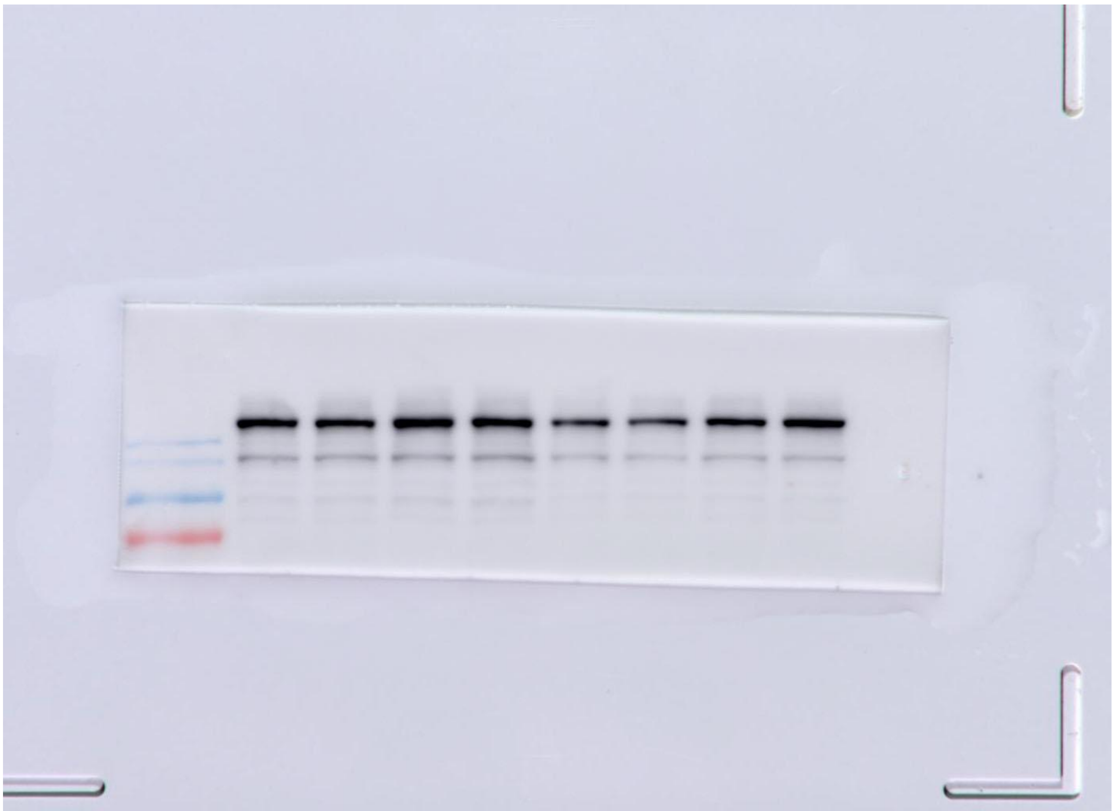

OCC

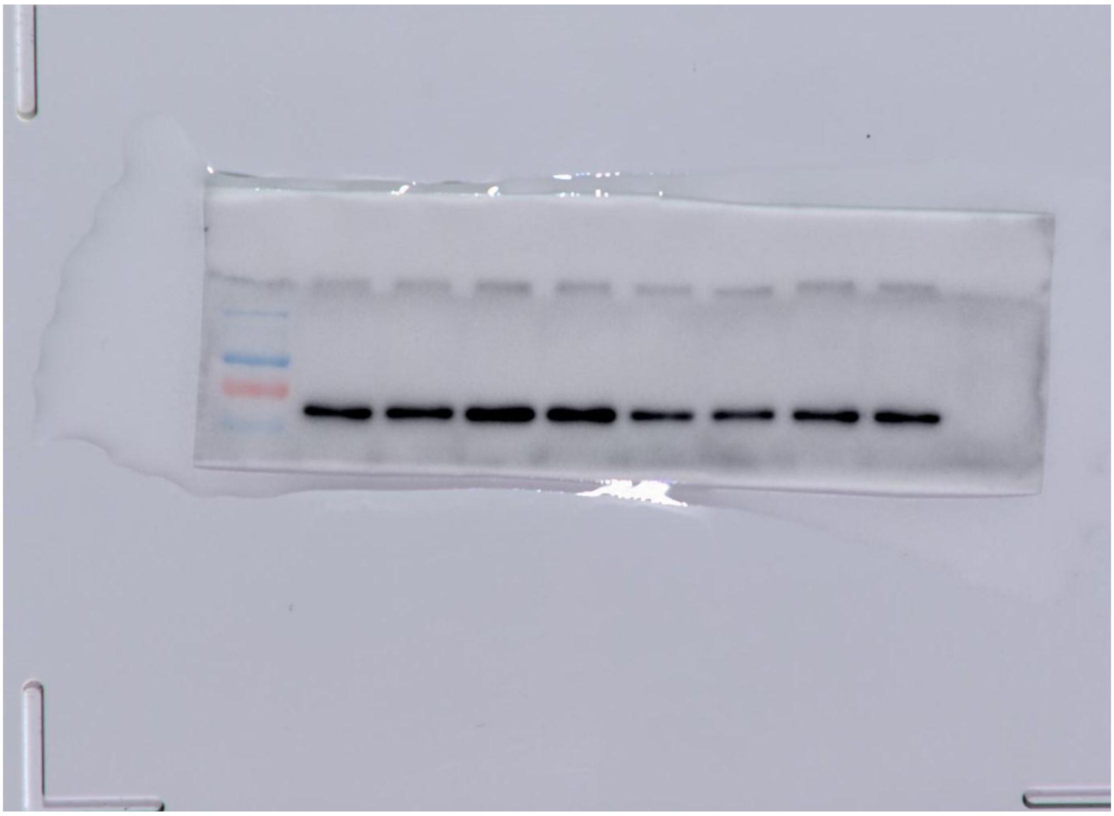

GAPDH

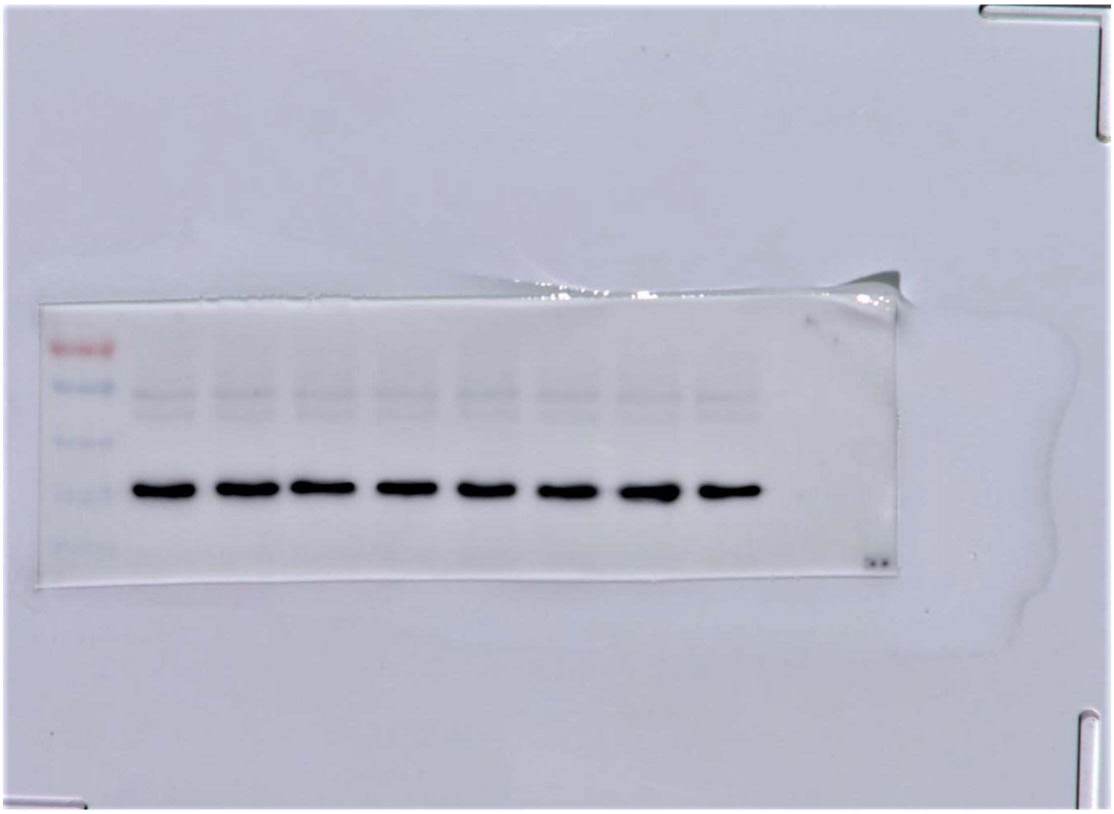

NLRP3

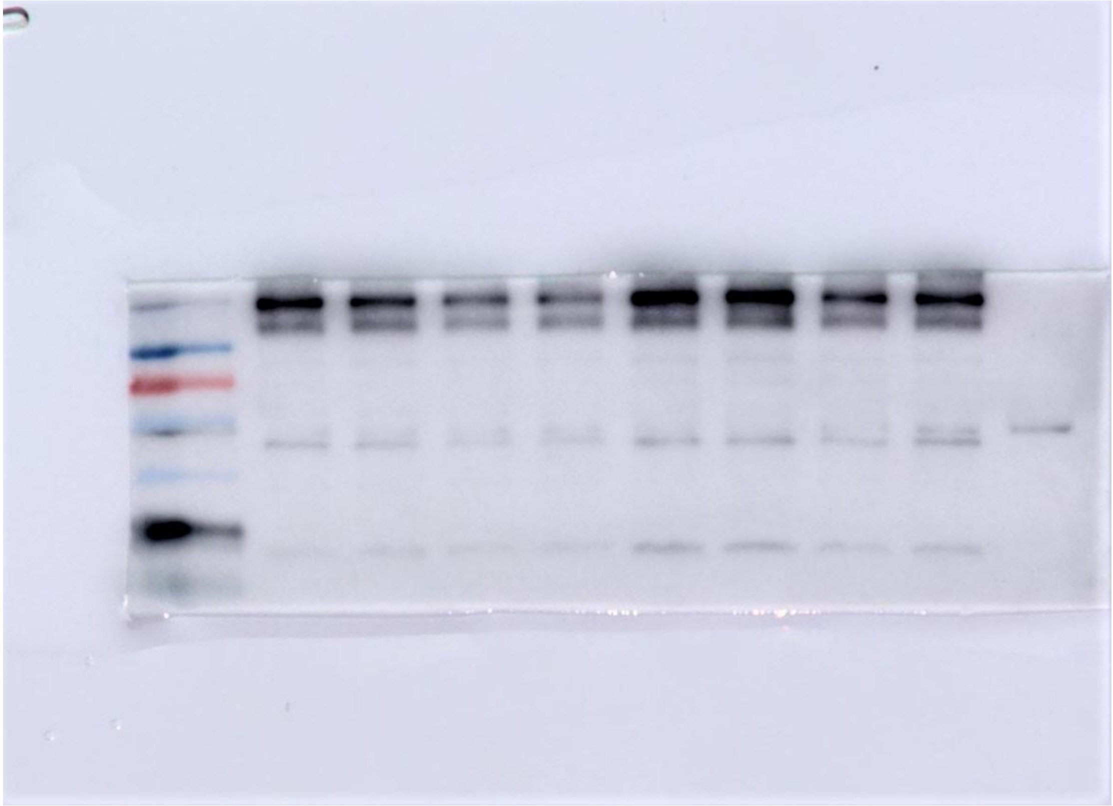

GSDMD/GSDMD-N

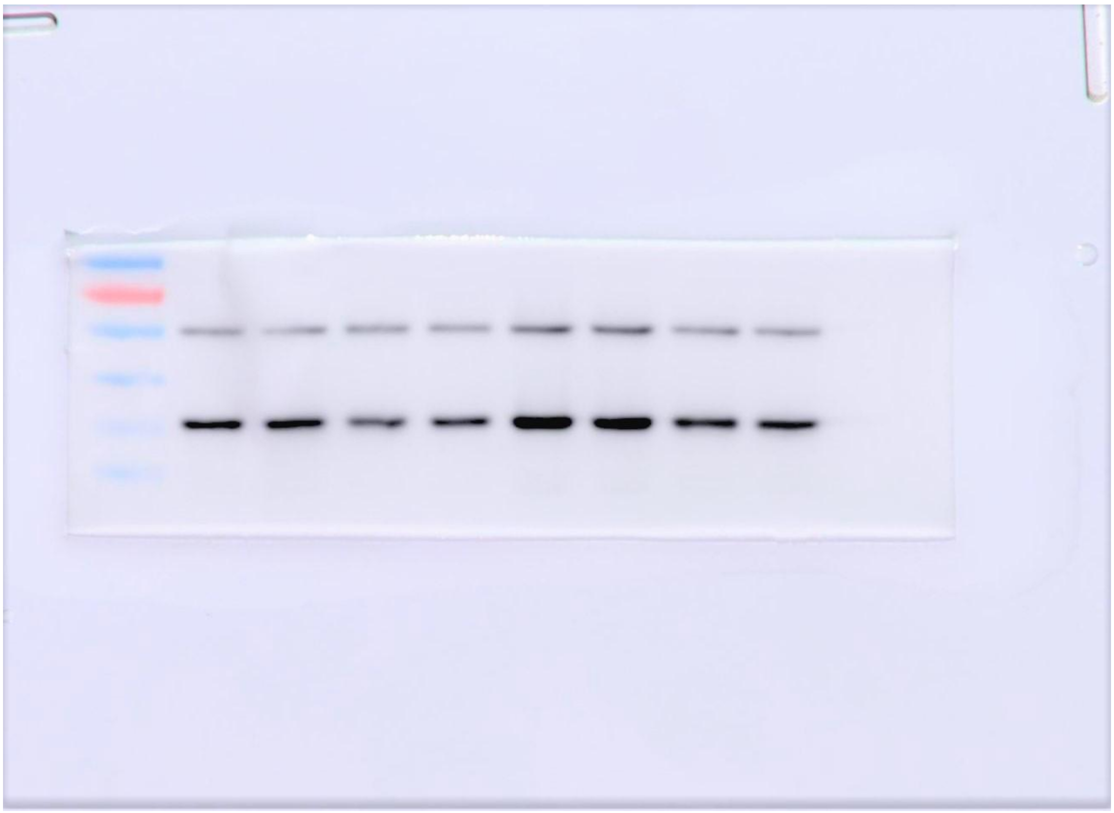

Caspase-1/ Caspase-1-p10

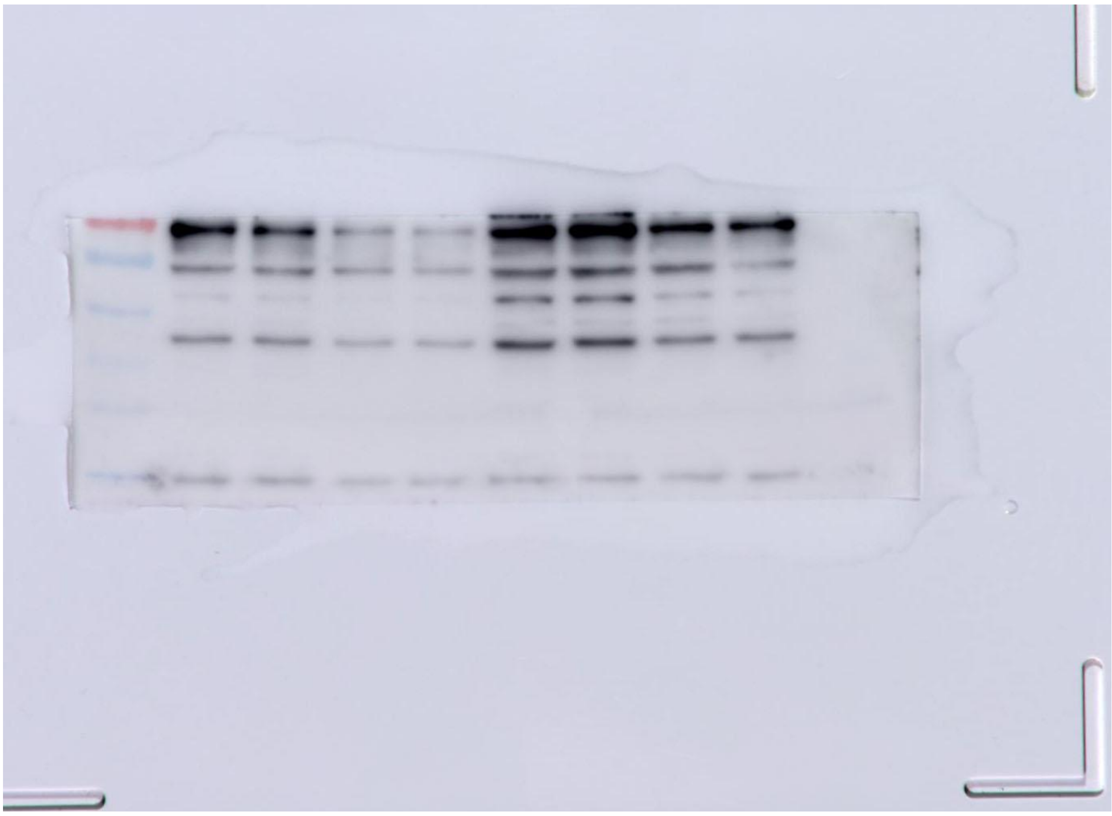

GAPDH

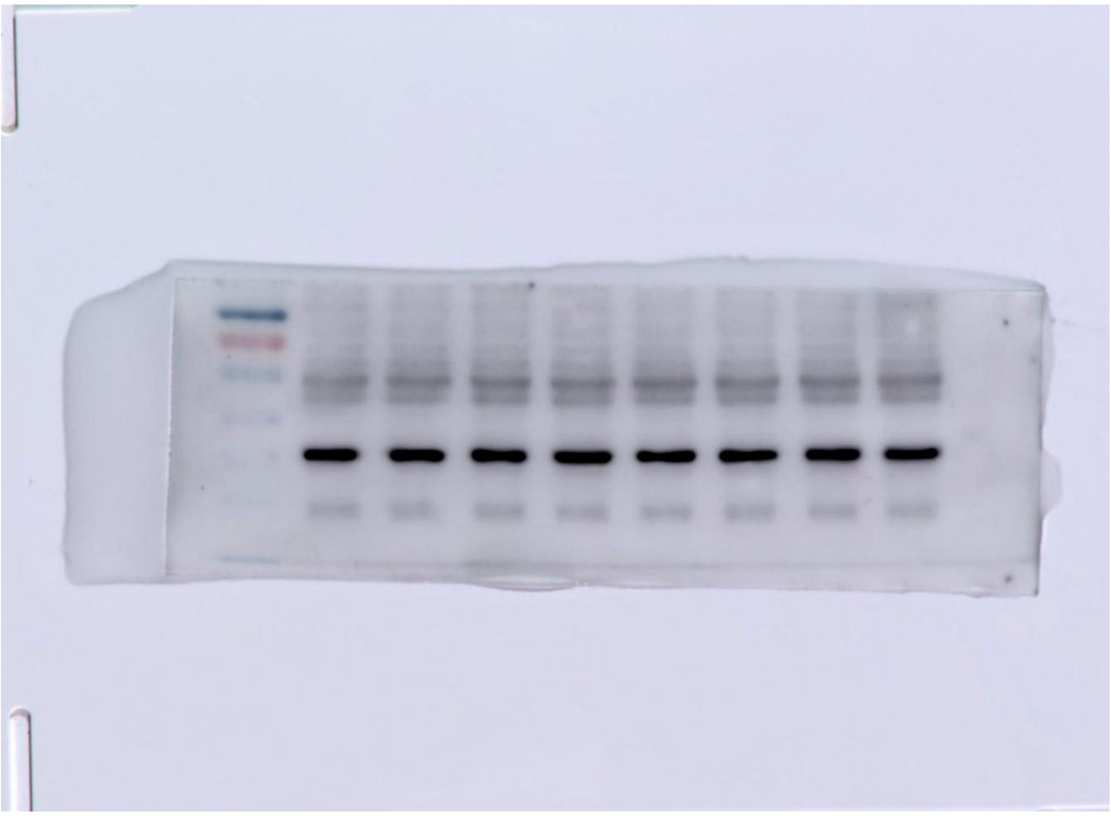

Fig 6

AMD3100: Zo-1

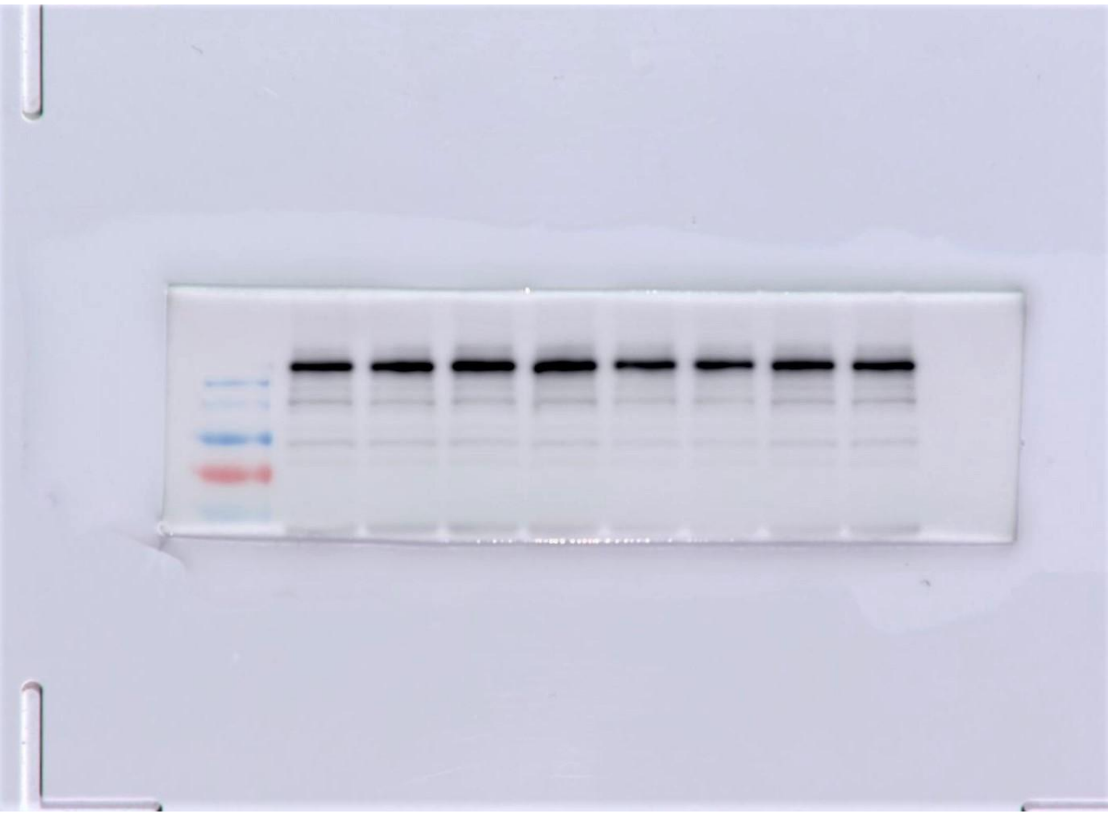

OCC

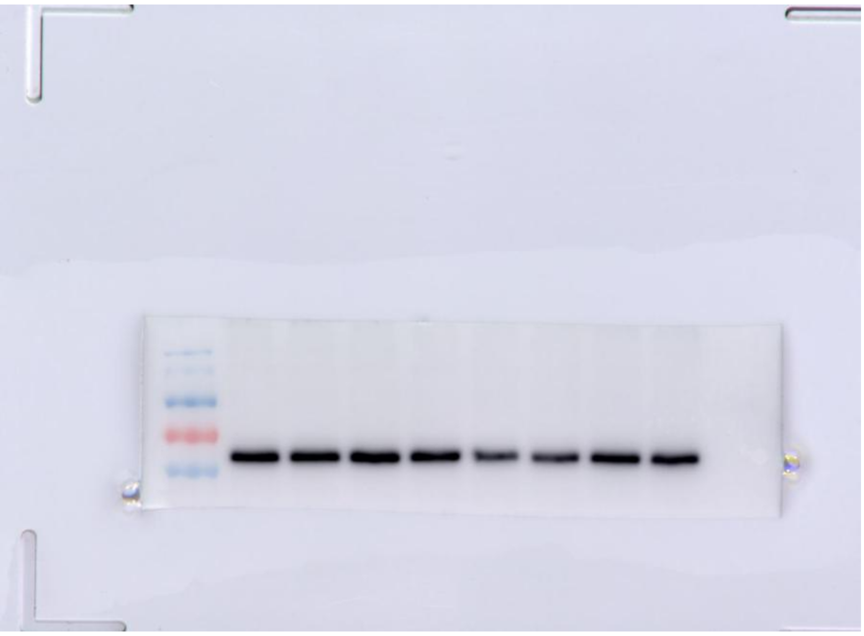

NLRP3

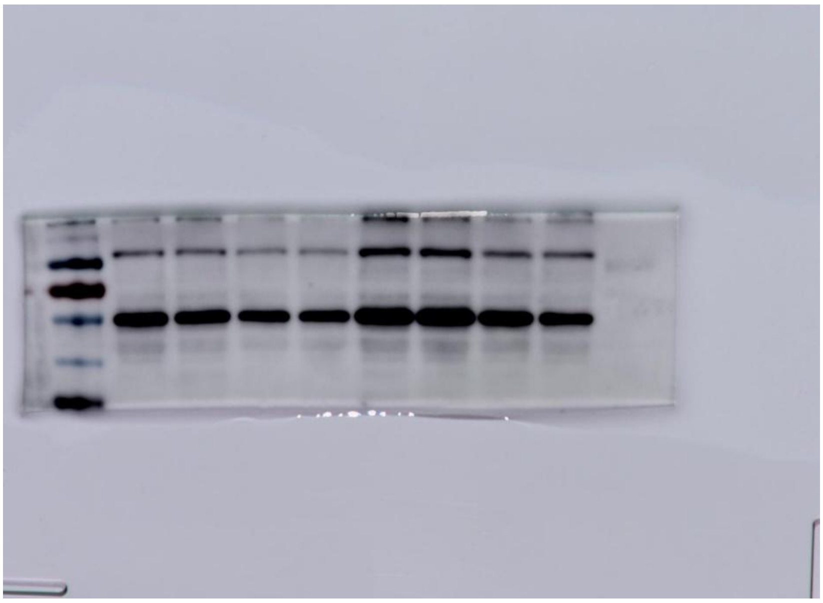

GSDMD/GSDMD-N

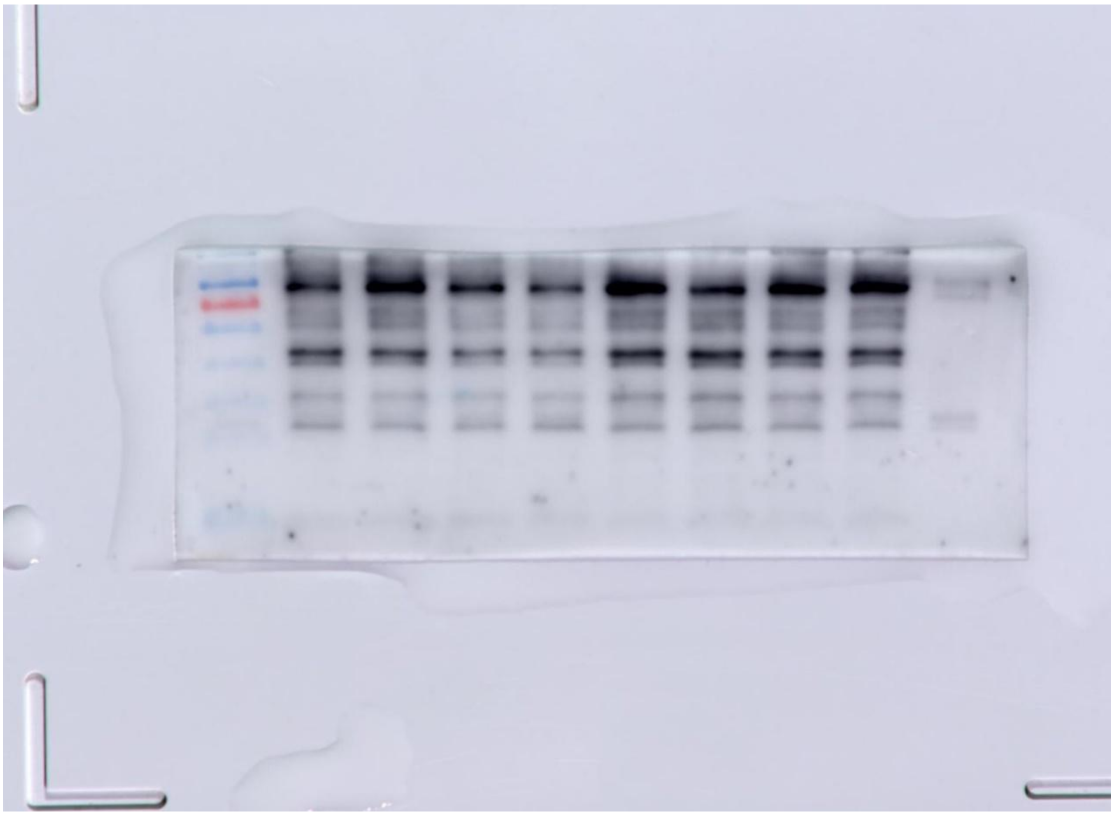

Caspase-1/ Caspase-1-p10

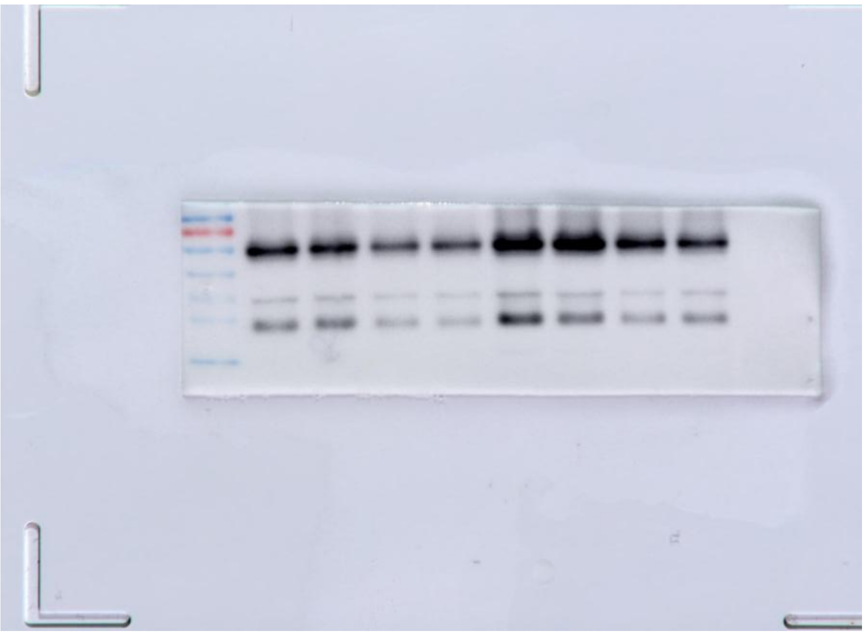

p-p65

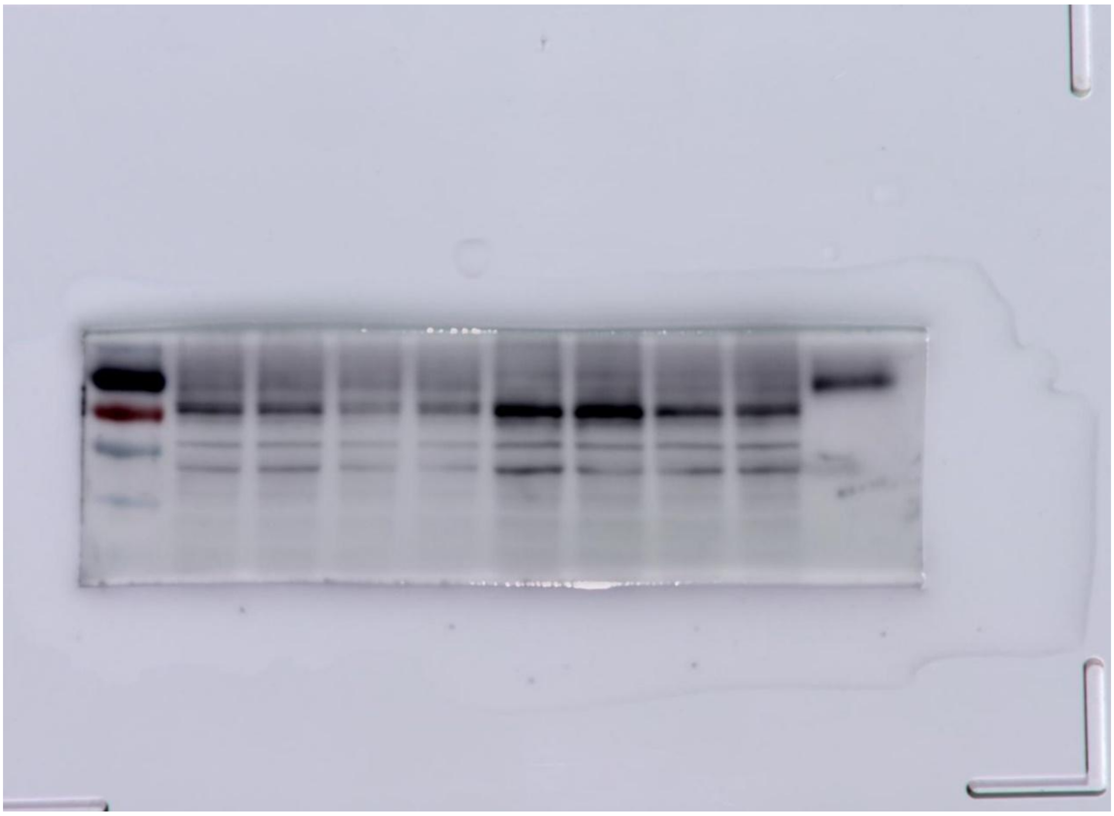

GAPDH

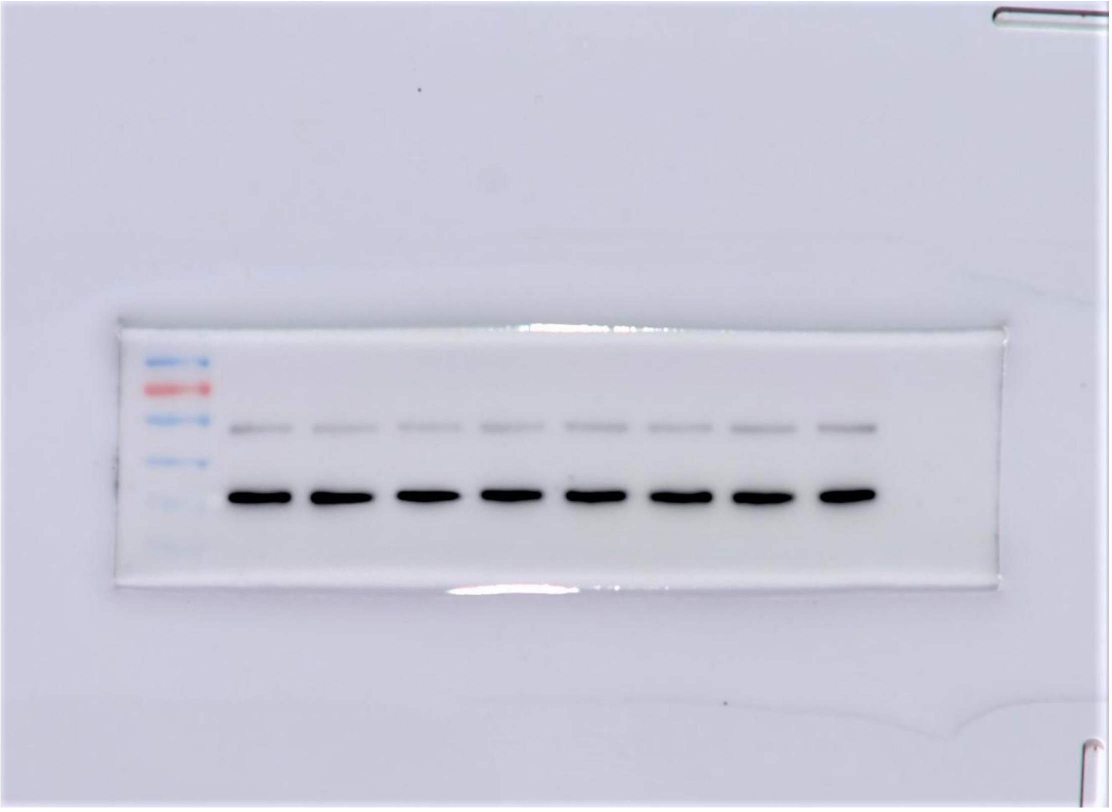

NUCC-390: ZO-1

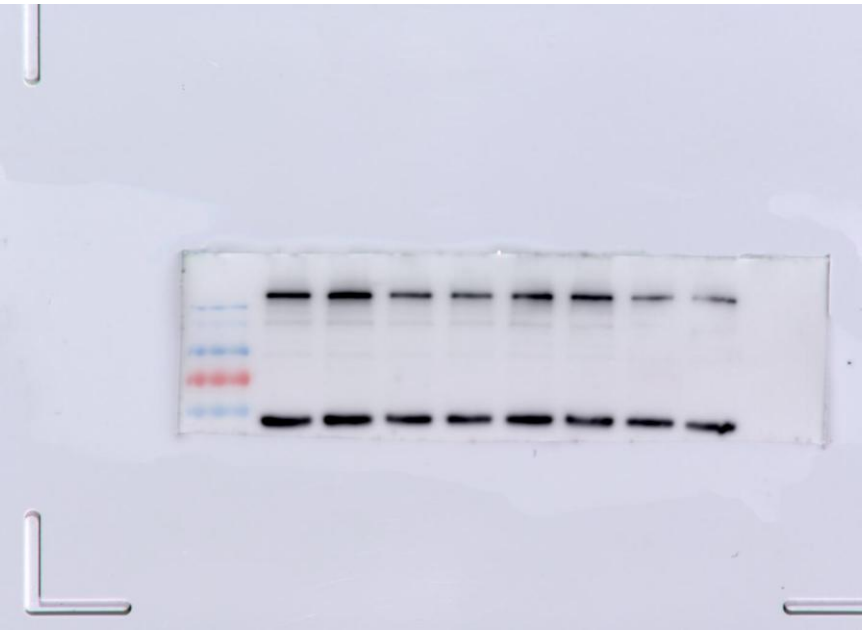

OCC

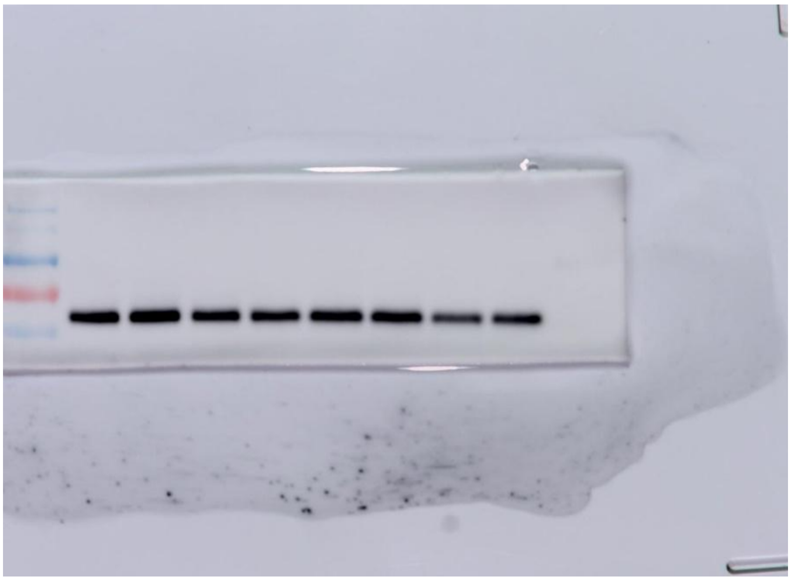

NLRP3

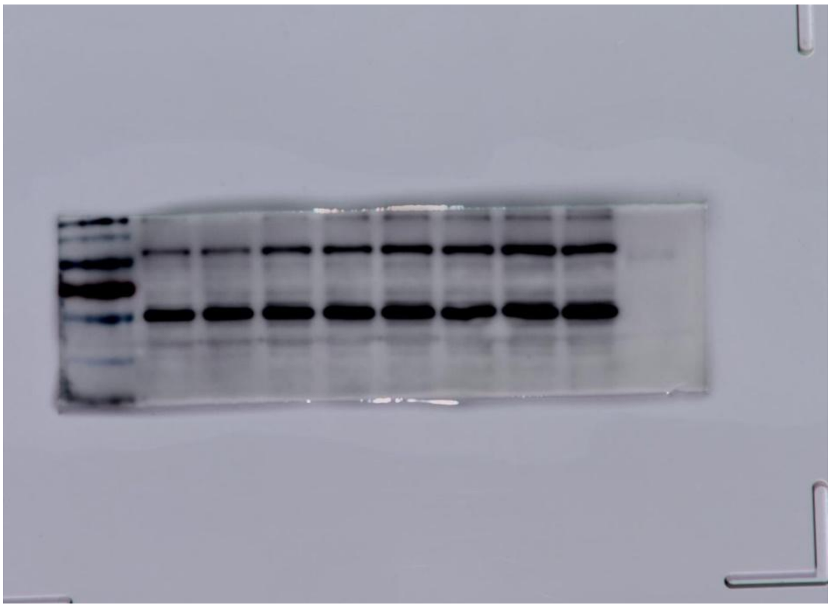

GSDMD/GSDMD-N

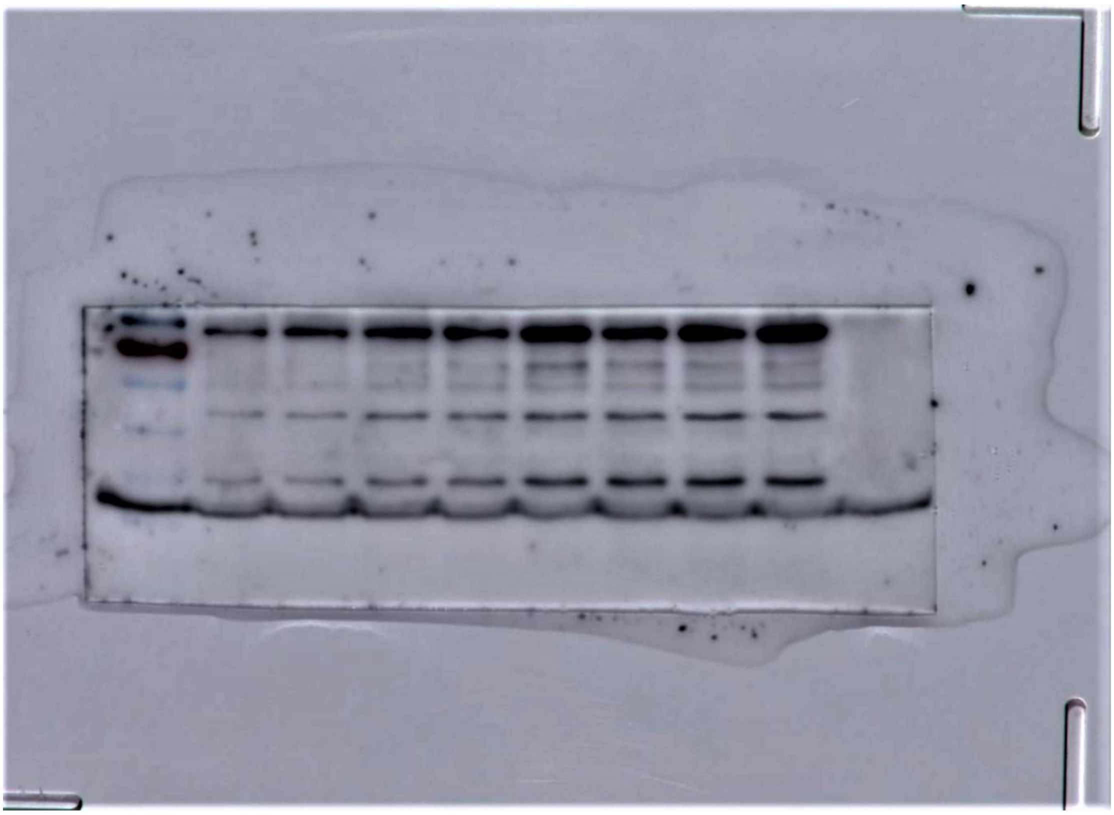

Caspase-1/ Caspase-1-p10

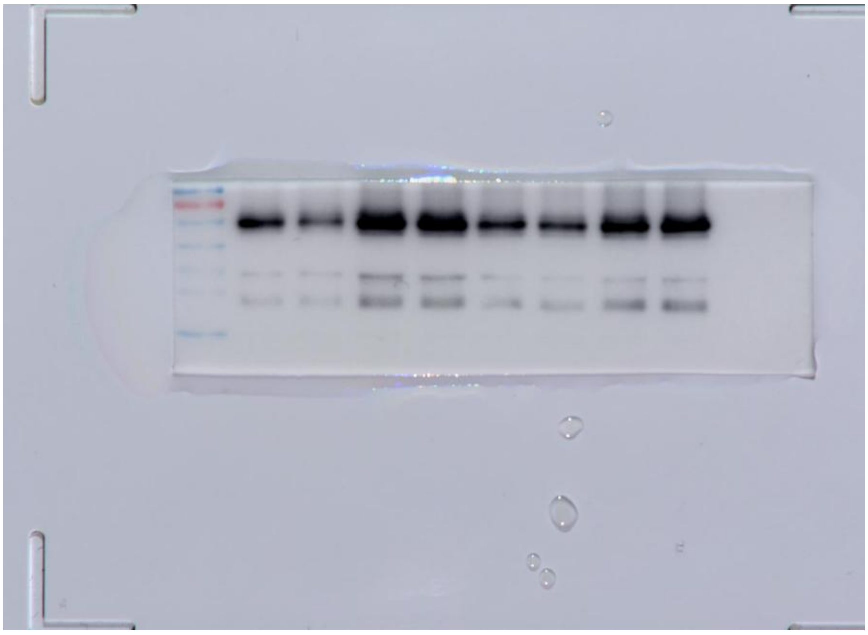

p-p65

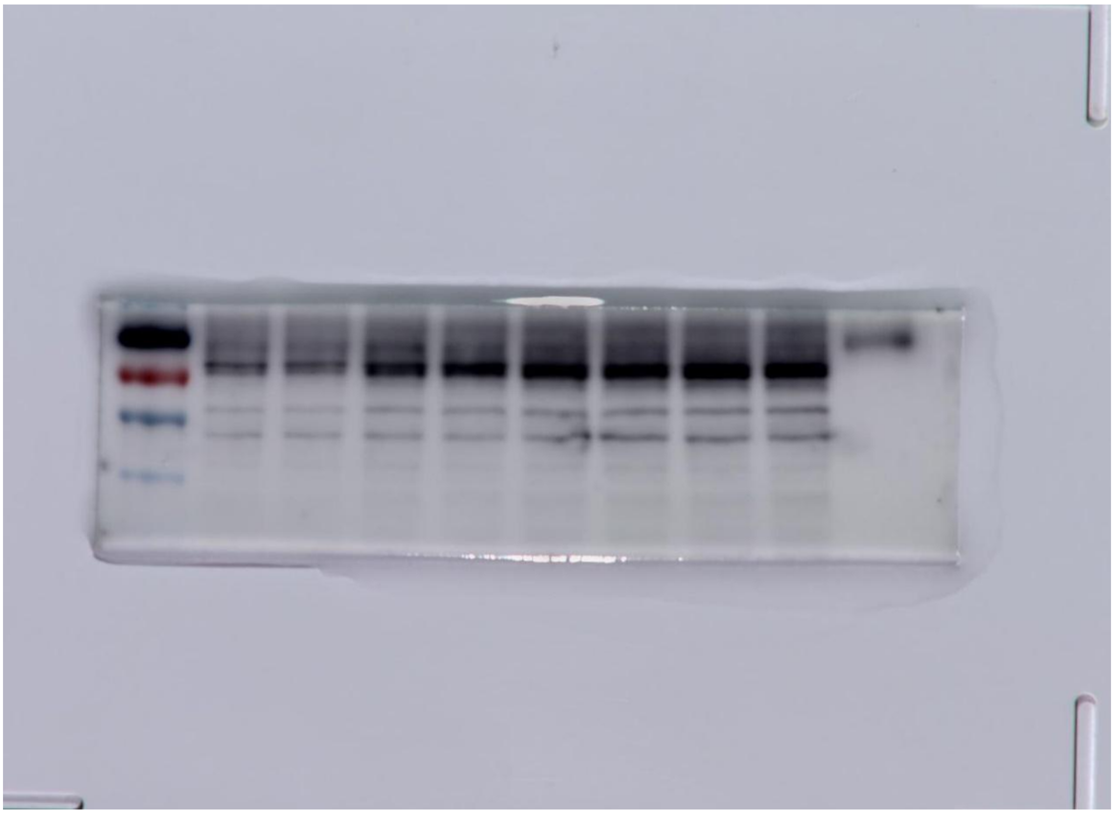

GAPDH

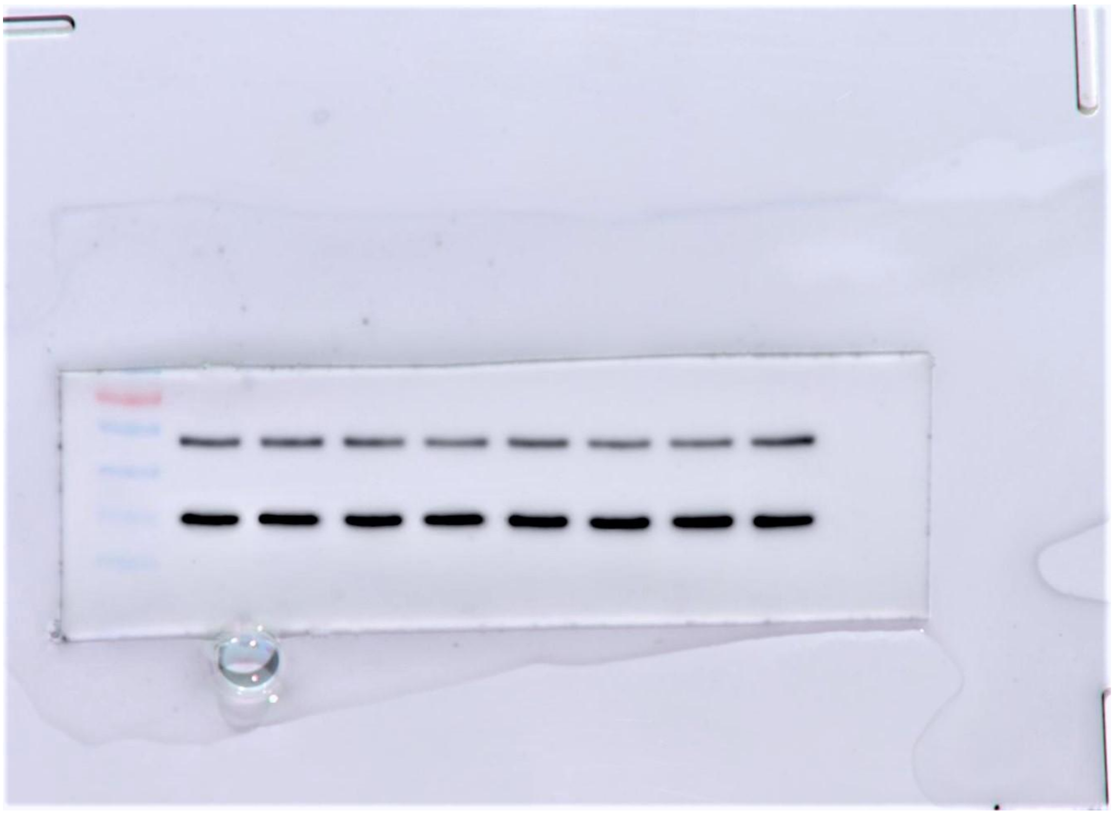

Fig7

Zo-1

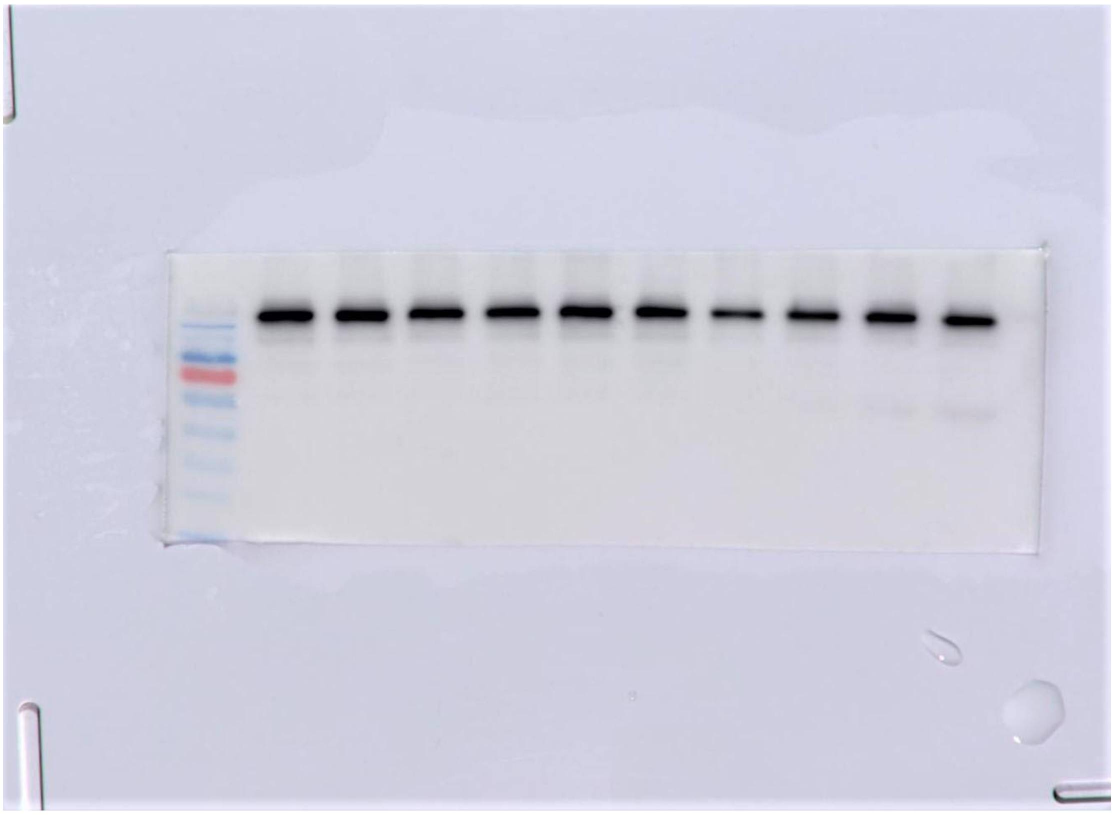

OCC

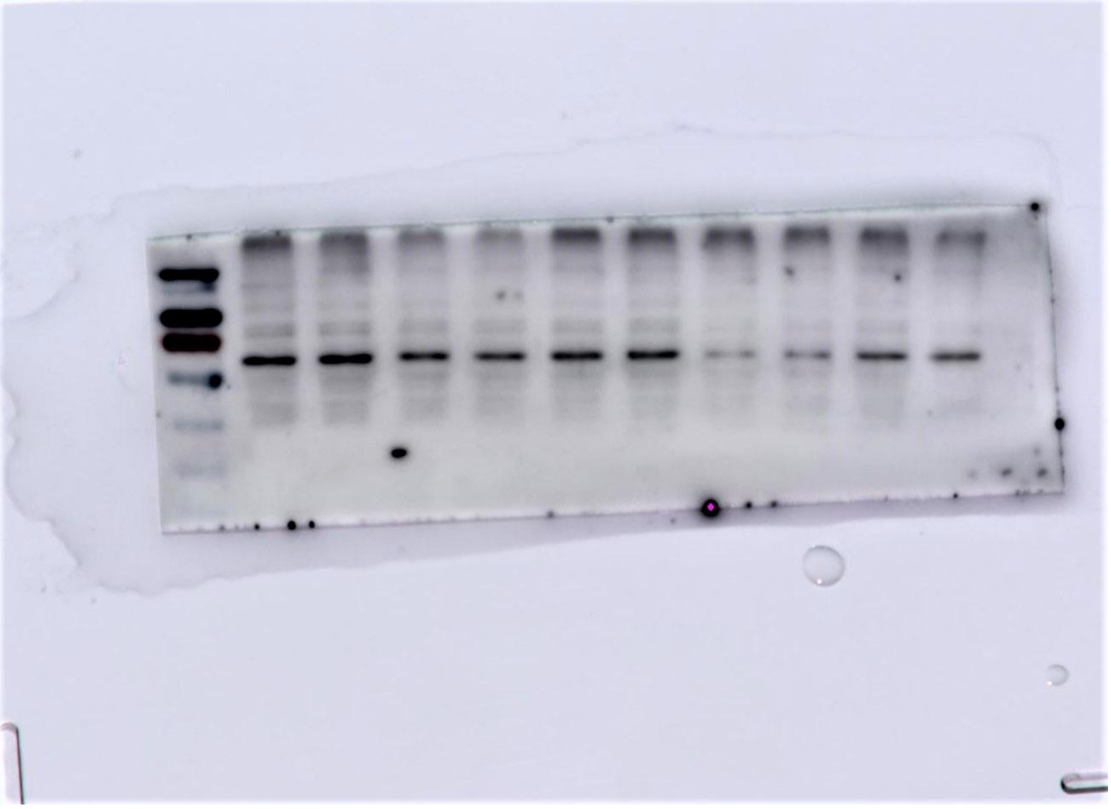

GAPDH

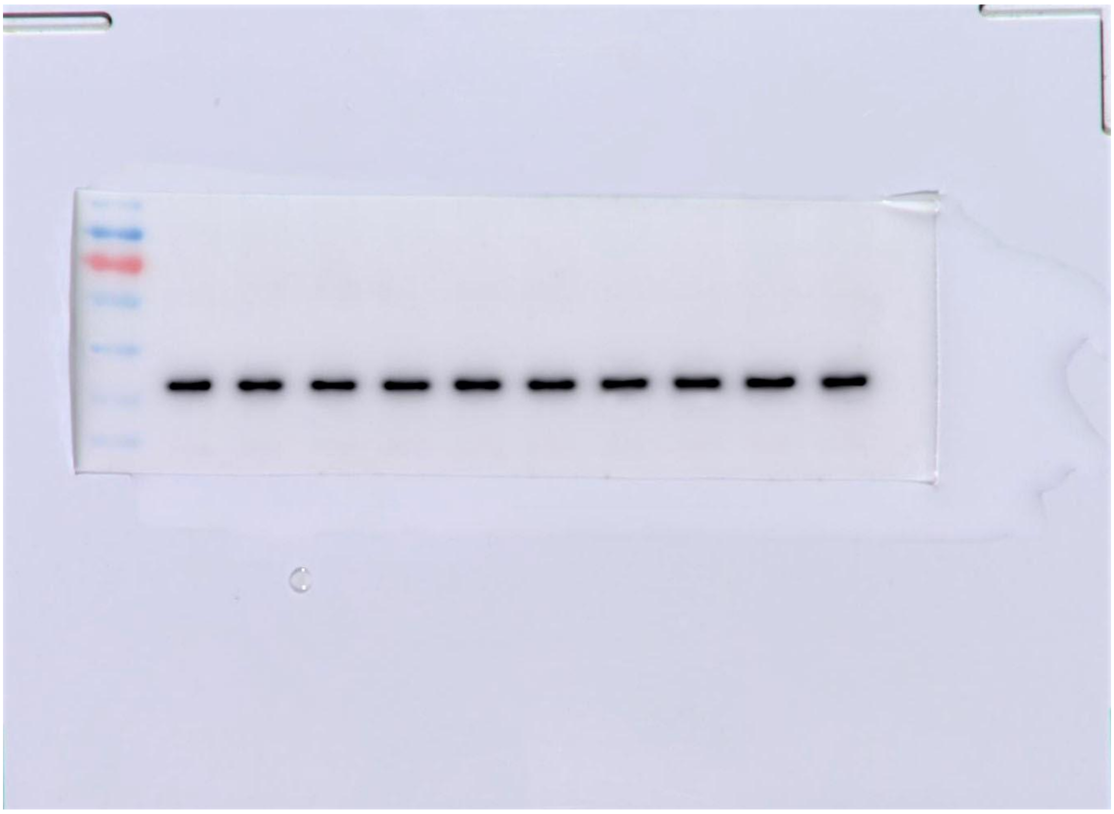

NLRP3

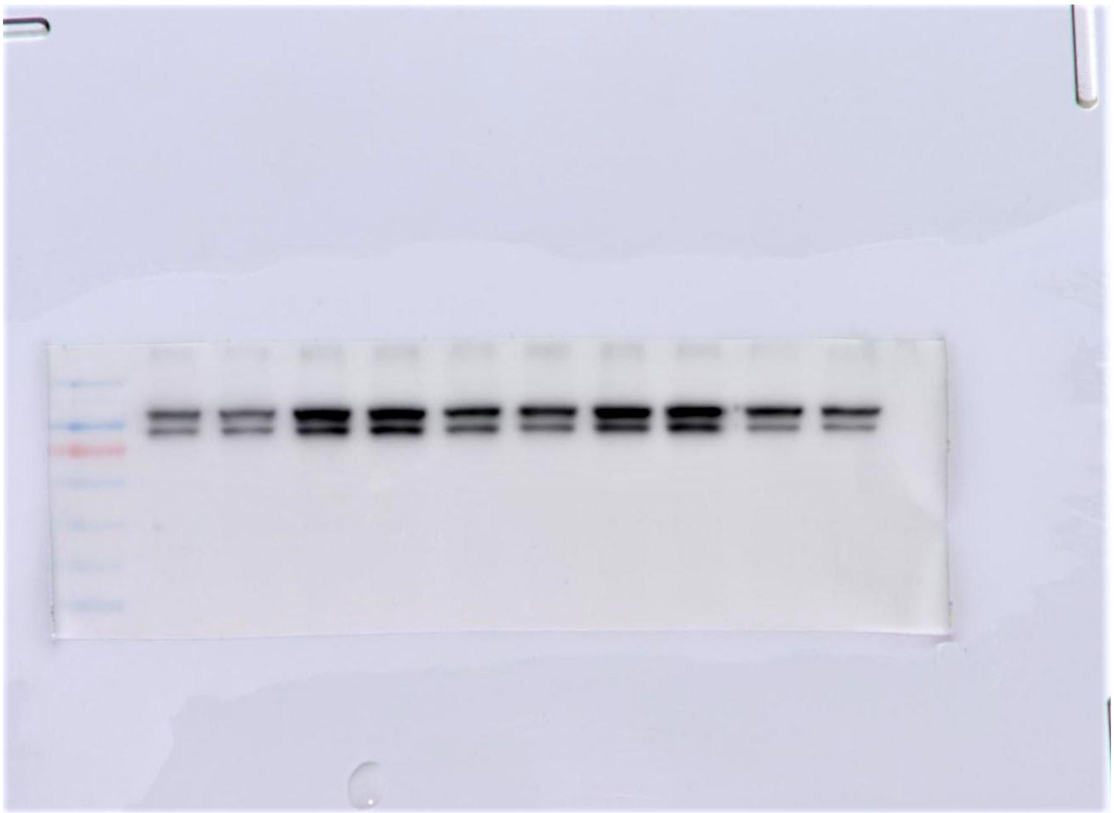

GSDMD/GSDMD-N

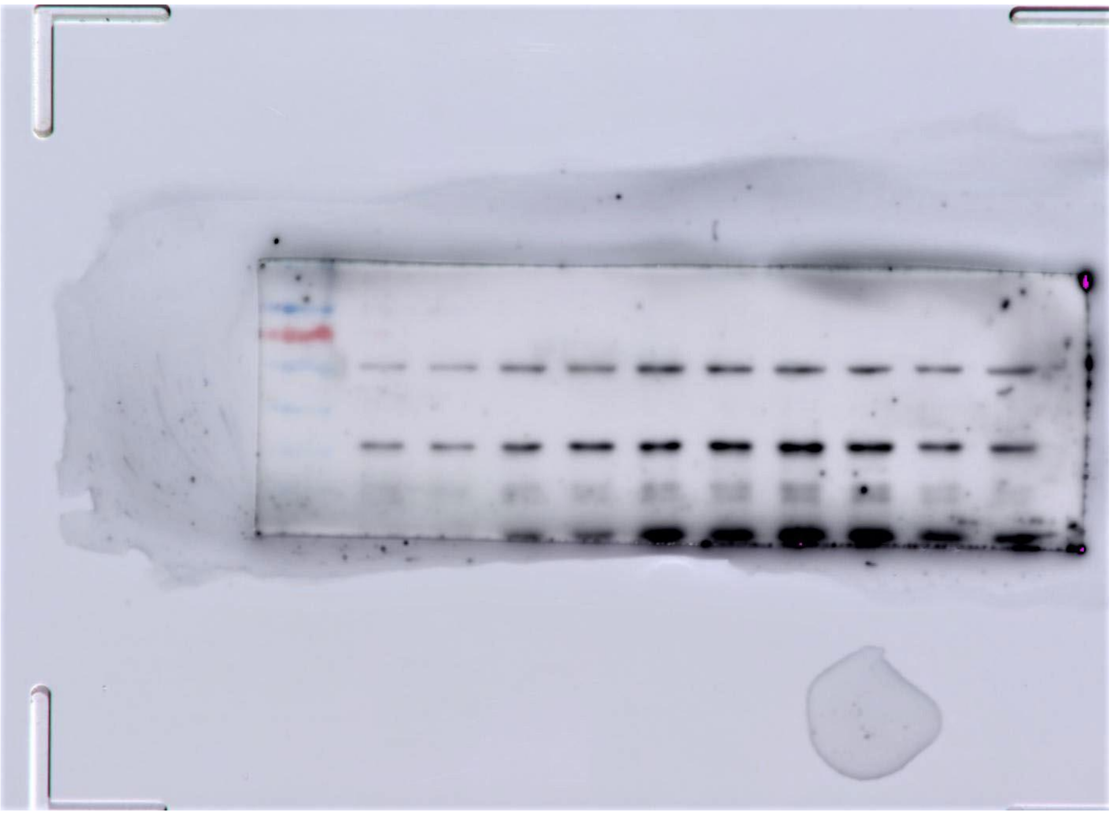

Caspase-1/ Caspase-1-p10

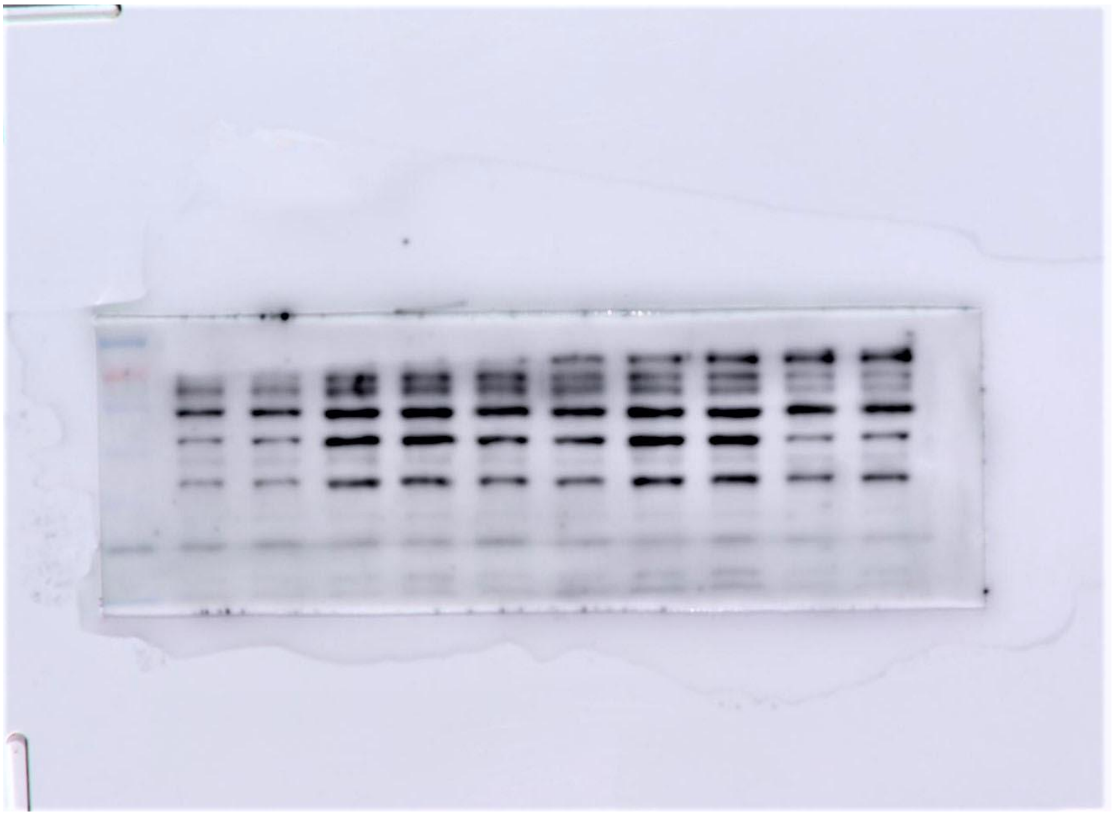

p-p65

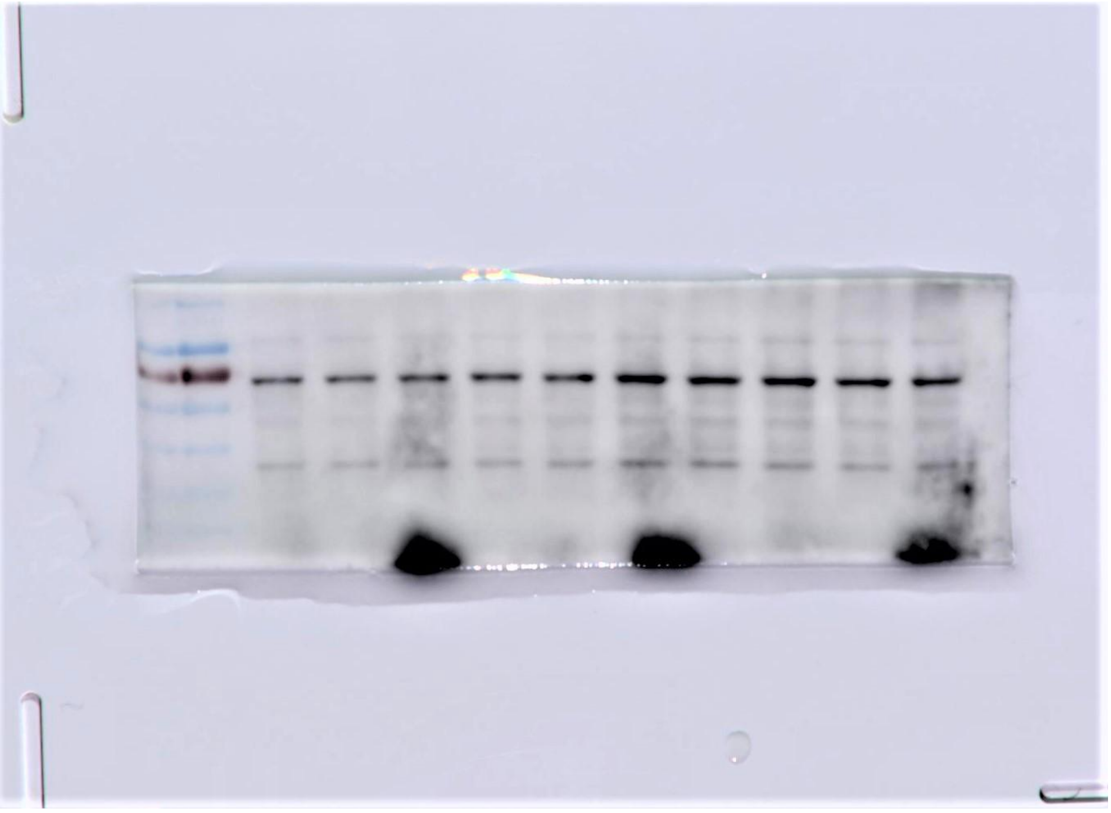

GAPDH

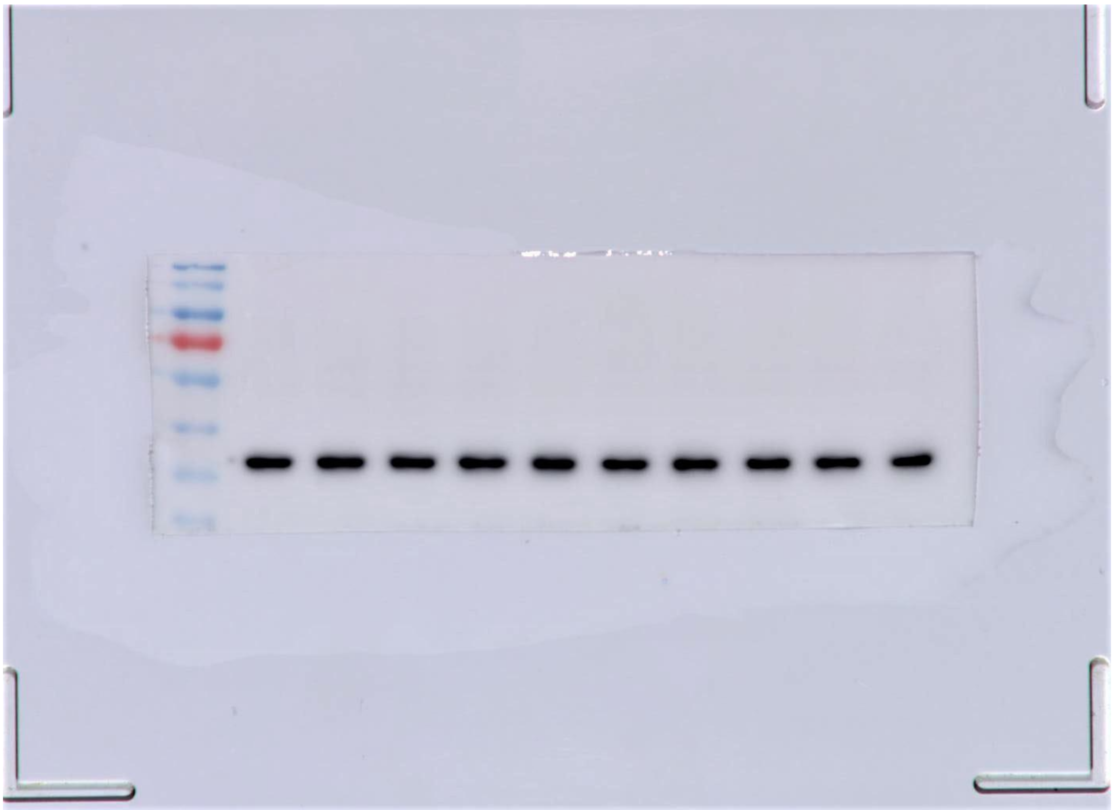

Fig8

ZO-1

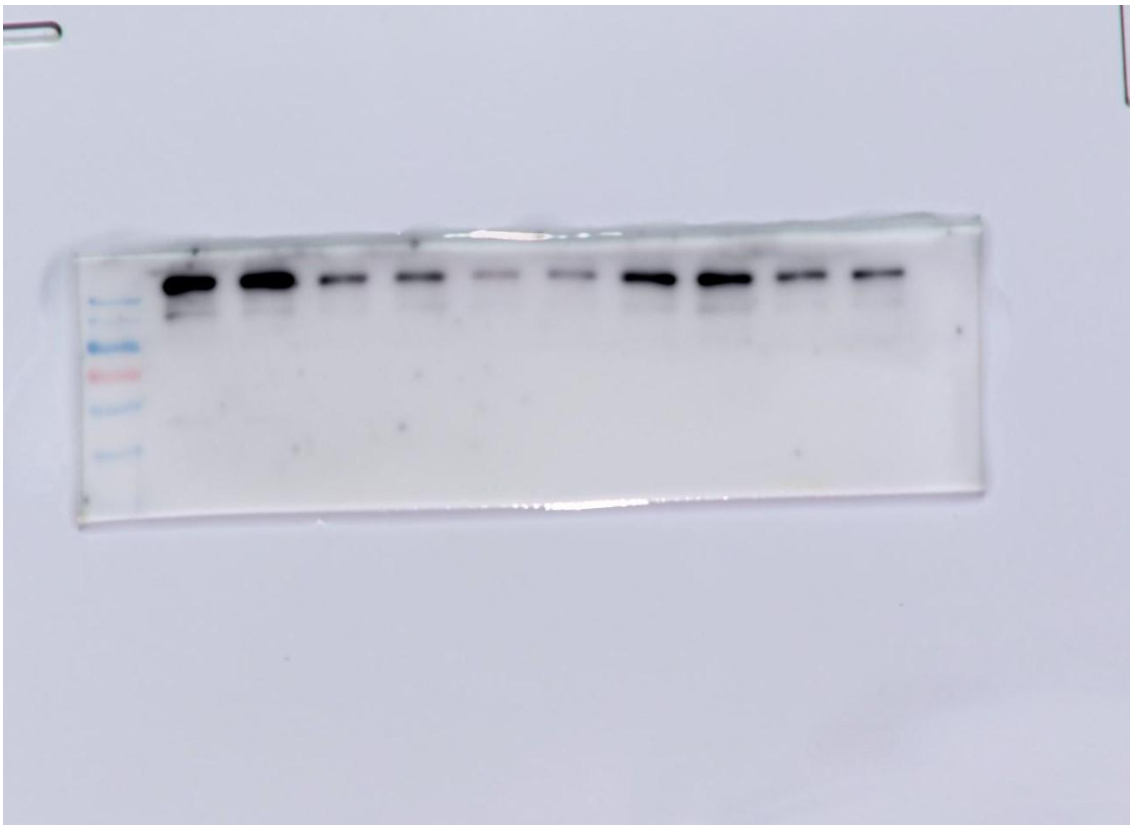

OCC

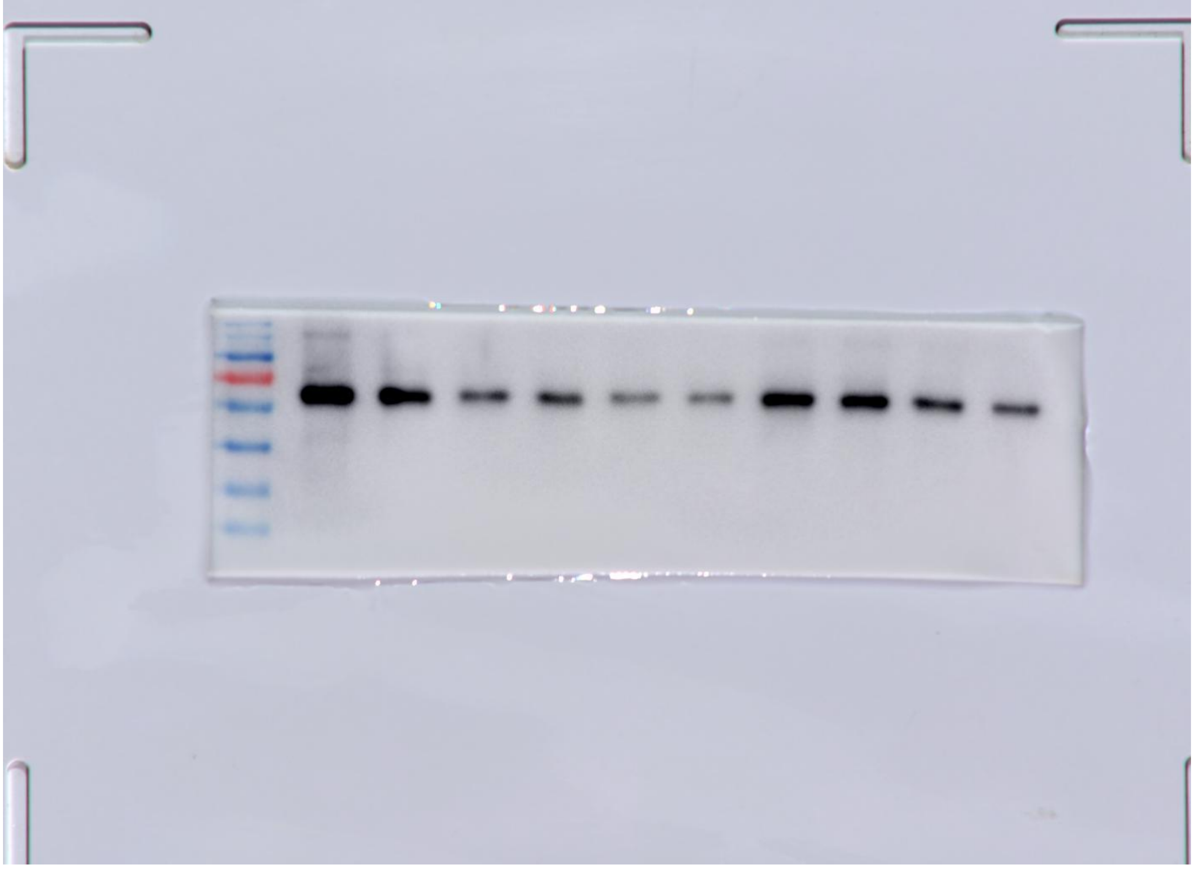

NLRP3

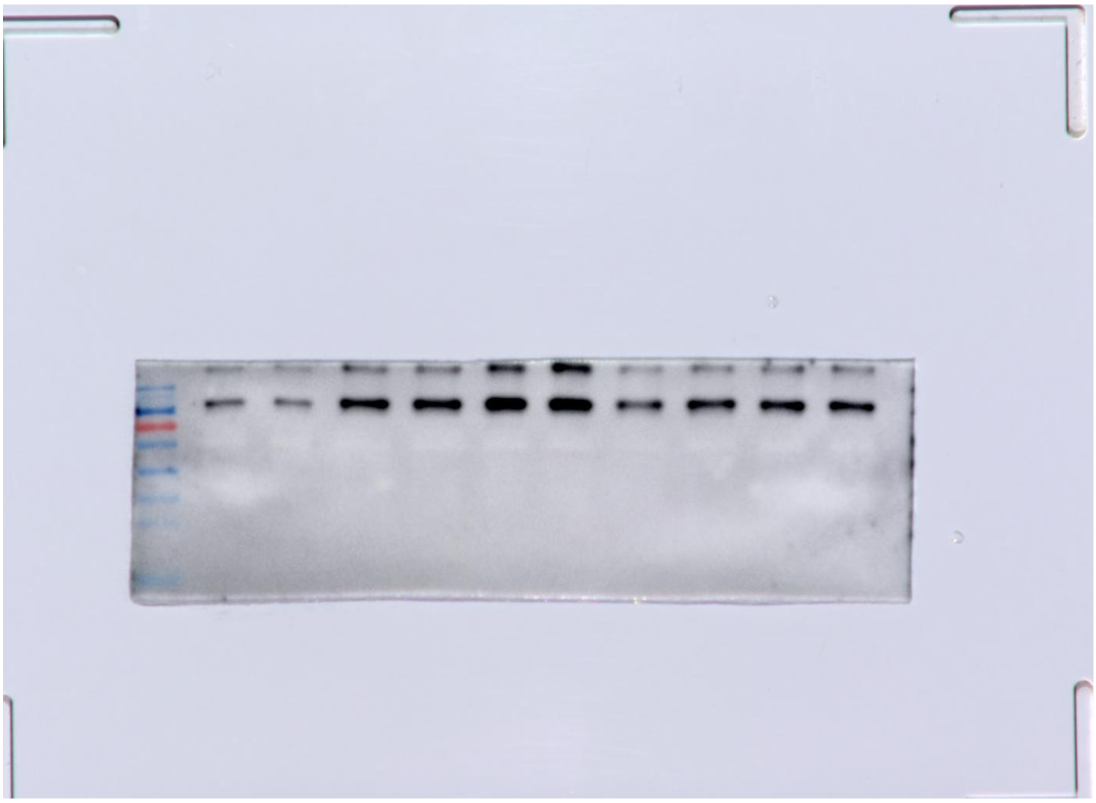

GSDMD、GSDMD-NT

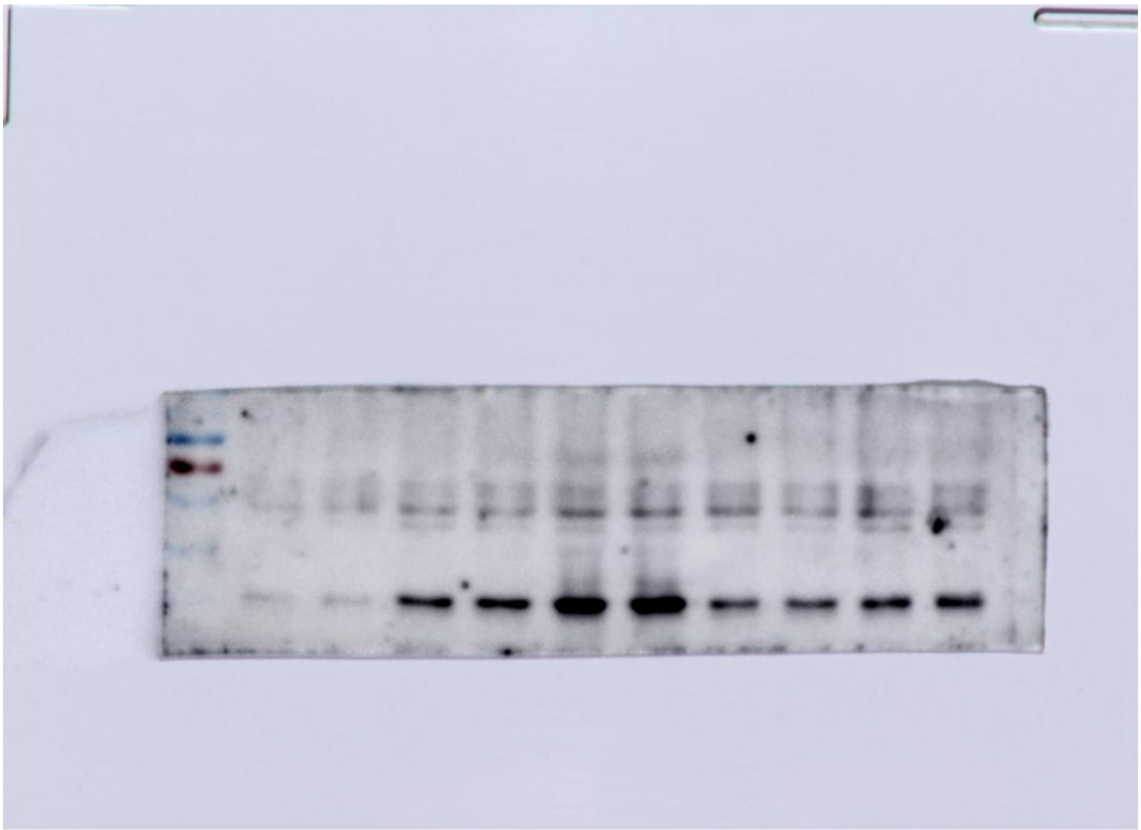

Pro-Caspase-1、Caspase-1-p10

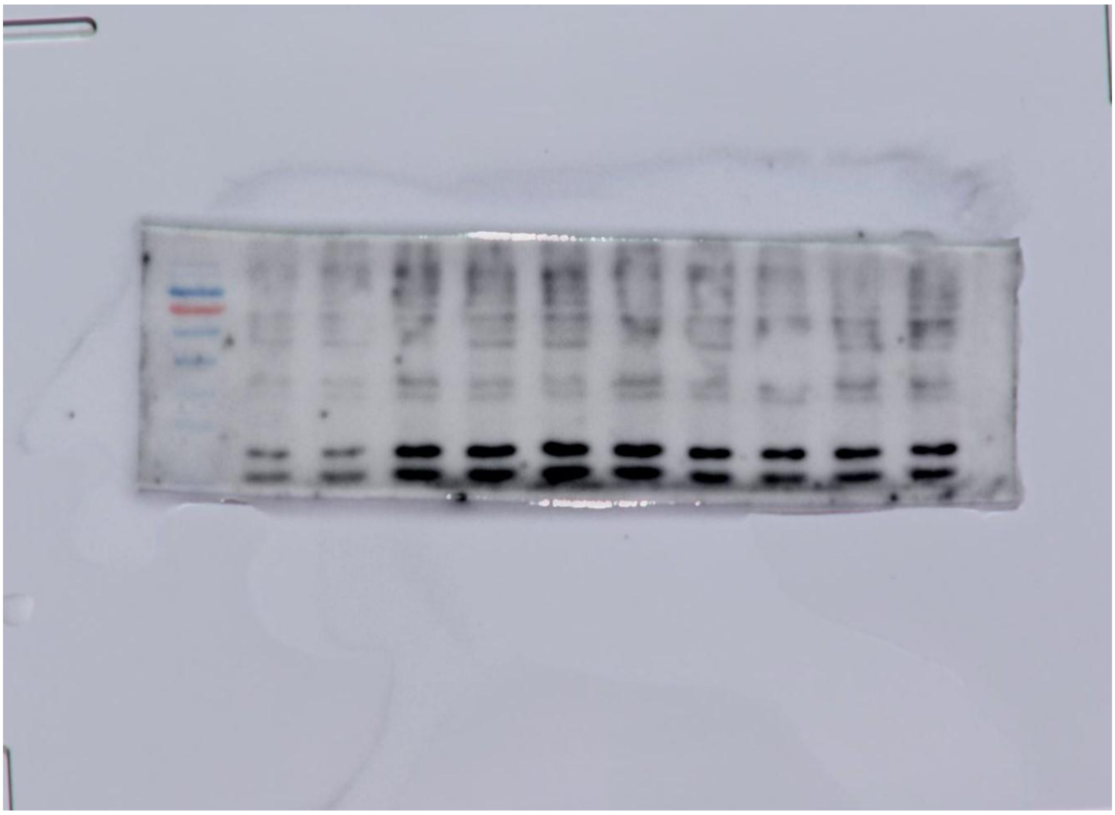

p-p65

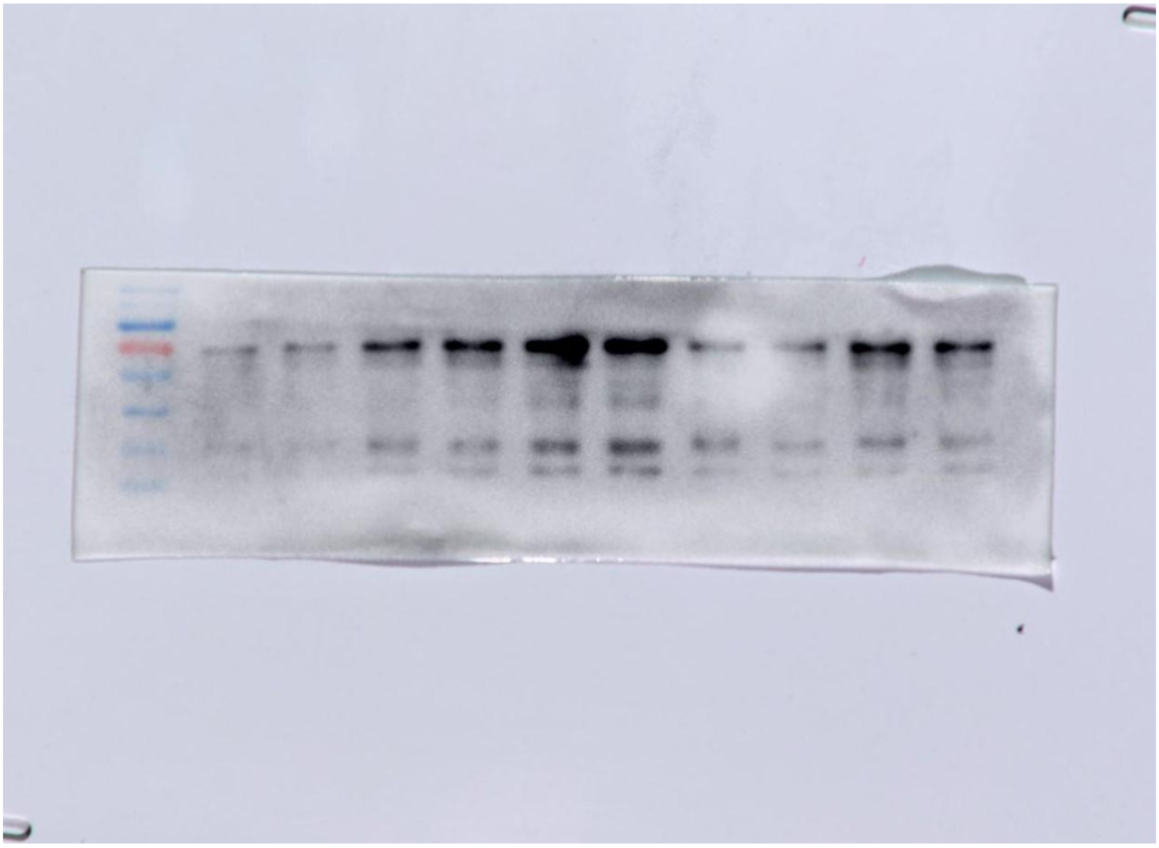

GAPDH

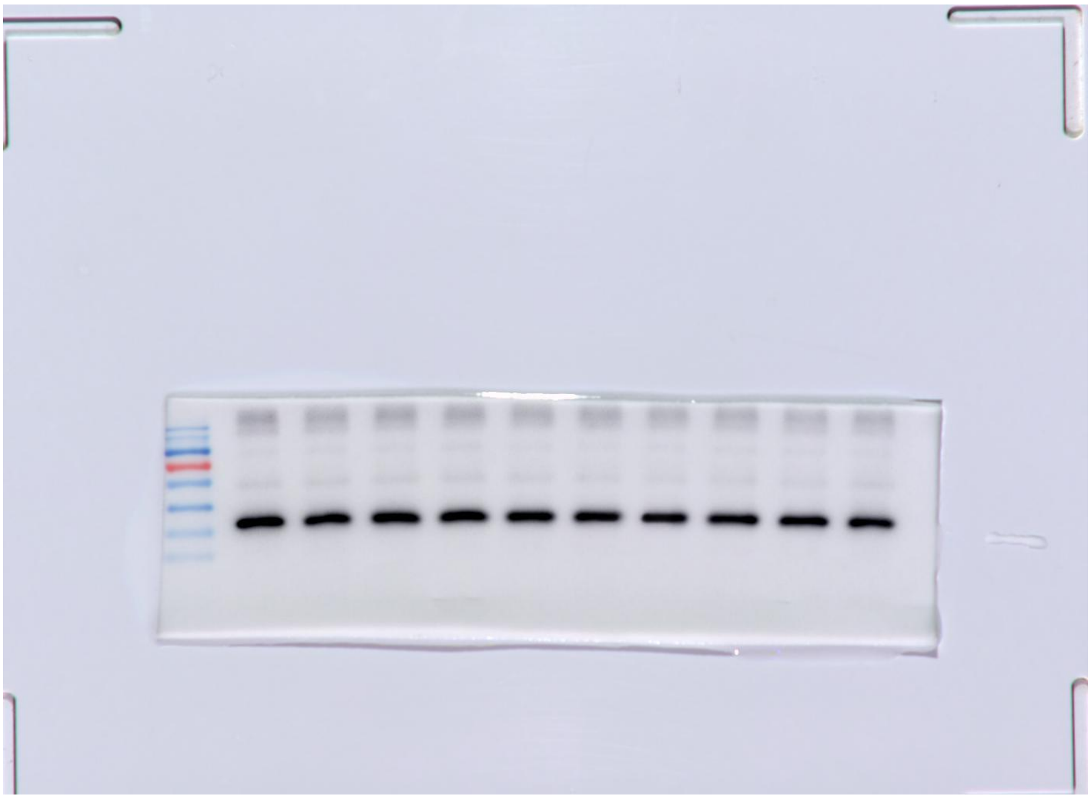

Supplement: Supplementary file 1 — Supplementary Material 1 [file 10753_2026_2494_MOESM1_ESM.pdf]
